# Machine learning in cardiovascular radiology: ESCR position statement on design requirements, quality assessment, current applications, opportunities, and challenges

**DOI:** 10.1007/s00330-020-07417-0

**Published:** 2020-11-19

**Authors:** Thomas Weikert, Marco Francone, Suhny Abbara, Bettina Baessler, Byoung Wook Choi, Matthias Gutberlet, Elizabeth M. Hecht, Christian Loewe, Elie Mousseaux, Luigi Natale, Konstantin Nikolaou, Karen G. Ordovas, Charles Peebles, Claudia Prieto, Rodrigo Salgado, Birgitta Velthuis, Rozemarijn Vliegenthart, Jens Bremerich, Tim Leiner

**Affiliations:** 1 Department of Radiology, University Hospital Basel, University of Basel, Petersgraben 4, 4031 Basel, Switzerland; 2 Department of Radiological, Oncological and Pathological Sciences, Sapienza University of Rome, Policlinico Umberto I, V.le Regina Elena 324, 00161 Rome, Italy; 3 Department of Radiology, UT Southwestern Medical Center, 5323 Harry Hines Boulevard, Dallas, TX 75390-9316 USA; 4 Institute of Diagnostic and Interventional Radiology, University Hospital Zurich, Raemistrasse 100, 8091 Zurich, Switzerland; 5 Radiology Department, Research Institute of Radiological Science, Center for Clinical Imaging Data Science, Yonsei University College of Medicine, 50-1 Yonsei-ro, Seodaemun-gu, Seoul, 03722 South Korea; 6 Department of Diagnostic and Interventional Radiology, Heart Center Leipzig – University Leipzig, Strümpellstrasse 39, 04289 Leipzig, Germany; 7 Department of Radiology, Weill Cornell Medicine, 520 East 70th Street, New York, NY 10021 USA; 8 Division of Cardiovascular and Interventional Radiology, Department of Bioimaging and Image-Guided Therapy, Medical University Vienna, Waehringer Guertel 18-20, A-1090 Vienna, Austria; 9 Department of Radiology, Hôpital Européen Georges Pompidou, APHP, University of Paris & INSERM, U970 29 rue Leblanc, 75015 Paris, France; 10 Radiological and Haematological Sciences Department, Fondazione Policlinico Universitario A. Gemelli- IRCCS, Università Cattolica S. Cuore, Largo Agostino Gemelli 8, 00168 Rome, Italy; 11 Department of Diagnostic and Interventional Radiology, University Hospital Tuebingen, Hoppe-Seyler-Strasse 3, 72076 Tübingen, Germany; 12 Department of Radiology and Biomedical Imaging, University of California- San Francisco, 505 Parnassus Ave, M396 Box 0628, San Francisco, CA 94143-0628 USA; 13 Department of Radiology, University Hospital Southampton, Tremona Road, Southampton, SO16 6YD UK; 14 School of Biomedical Engineering and Imaging Sciences, King’s College London, St Thomas’ Hospital, London, SE1 7EH UK; 15 Department of Radiology, Antwerp University Hospital & Holy Heart Hospital Lier, Wilrijkstraat 10, 2650 Edegem, Belgium; 16 Department of Radiology, Utrecht University Medical Center, Heidelberglaan 100, 3584 CX Utrecht, The Netherlands; 17 Department of Radiology, University of Groningen, University Medical Center Groningen, Hanzeplein 1, 9713 GZ Groningen, The Netherlands

**Keywords:** Artificial intelligence, Machine learning, Diagnostic techniques, cardiovascular, Radiology, Consensus

## Abstract

**Abstract:**

Machine learning offers great opportunities to streamline and improve clinical care from the perspective of cardiac imagers, patients, and the industry and is a very active scientific research field. In light of these advances, the European Society of Cardiovascular Radiology (ESCR), a non-profit medical society dedicated to advancing cardiovascular radiology, has assembled a position statement regarding the use of machine learning (ML) in cardiovascular imaging. The purpose of this statement is to provide guidance on requirements for successful development and implementation of ML applications in cardiovascular imaging. In particular, recommendations on how to adequately design ML studies and how to report and interpret their results are provided. Finally, we identify opportunities and challenges ahead. While the focus of this position statement is ML development in cardiovascular imaging, most considerations are relevant to ML in radiology in general.

**Key Points:**

*• Development and clinical implementation of machine learning in cardiovascular imaging is a multidisciplinary pursuit.*

*• Based on existing study quality standard frameworks such as SPIRIT and STARD, we propose a list of quality criteria for ML studies in radiology.*

*• The cardiovascular imaging research community should strive for the compilation of multicenter datasets for the development, evaluation, and benchmarking of ML algorithms.*

**Supplementary Information:**

The online version contains supplementary material available at (10.1007/s00330-020-07417-0).

## Introduction

**Fig. 1:**
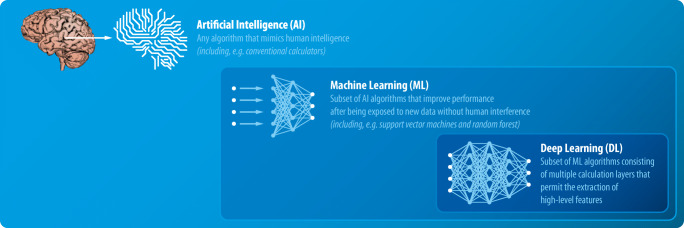
Relation and definition of artificial intelligence (AI), machine learning (ML), and deep learning (DL)

Artificial intelligence (AI), machine learning (ML), and deep learning (DL) are currently getting a lot of attention in the public arena and in science [1]. Their relation is hierarchically nested as shown in Fig. 1. AI is an umbrella term encompassing all techniques that mimic human intelligence, which have been studied and applied for decades [2], whereas ML describes a subset of AI algorithms that learn to map input parameters to output from training data (supervised ML) or identify previously undetected patterns (unsupervised ML). DL comprises a subset of ML algorithms that use multiple, connected calculation layers [3].

Open source programming tools, as well as greater computational power and easier data transfer facilitate the current boost in availability and productivity of AI algorithms. Concretely, in the most prevalent case of supervised ML, multiple pairs of input (e.g., MR image data of the heart) and output (e.g., ground truth segmentation of the left ventricle [LV]) are used for training. Subsequently, the trained algorithm can be used to automatically solve the learned task upon presentation of new, unseen input data. The fundamentals of ML have been described extensively elsewhere [4–6].

ML algorithms are of special interest to radiologists, because main areas of application are image processing, image analysis, and detection of findings—all core components of the radiological workflow before interpretation. One of their strengths is image segmentation, which is a prerequisite for analyses such as calculation of cardiac stroke volumes. Consequently, an increasing number of ML algorithms have been designed and evaluated in the field of cardiovascular radiology. Segmentation tasks are predominantly solved with DL algorithms, which have shown performances superior to traditional image processing methods. However, applications of ML extend beyond image analysis and can support many other tasks within the field of radiology such as triage of exams according to urgency or provision of a second reading to avoid missing relevant findings. They can also help with predicting outcomes and extending the diagnostic capabilities of CT and MRI, e.g., by assessing the fractional flow reserve from cardiac CT angiography.

In light of these advances, the European Society of Cardiovascular Radiology (ESCR), a non-profit medical society dedicated to advancing cardiovascular radiology, has assembled a position statement regarding the use of ML in cardiovascular imaging in close cooperation with other leading societies in the field. While the focus of this position statement is ML development in cardiovascular imaging, most considerations are relevant to ML in radiology in general.

## Requirements for successful development and implementation of ML algorithms in radiology

- Human resources and expertise

- Consensus statement
*•Machine learning projects in cardiovascular imaging should involve experts with different professional backgrounds, mainly medical and ML experts, and in later stages also experts in user interface design and regulatory matters.*
*•The research and business community should agree on common data format standards and easy export of segmentation masks from clinically used post-processing software is needed to foster data interchangeability and reusability of data.*
*•Integration of ML algorithms into existing clinical workflows should be smooth, preferably into primary systems (RIS/PACS), to assure utility and acceptance by users.*

A successful ML project in radiology is almost always multidisciplinary. It can be seen as a four-step process, each step requiring special expertise and ideally a close cooperation between multiple professionals, mainly medical and ML experts. This section describes human resources required for a successful ML project as well as key activities at each of the four steps.

The initial, crucial question is what problem to solve. For this, one needs clinical domain knowledge to identify a relevant problem. On the other hand, knowledge in programming is needed to assess whether the identified clinical problem is technically solvable by means of ML. Furthermore, one should always ask whether ML is the best solution for the problem or if there are other, less complex solutions, such as a manual workflow in the case of rarely occurring tasks. Apart from ML, there are many traditional imaging processing techniques like region growing that are very effective for e.g. tracing the coronary arteries [7]. To clarify these issues, a close cooperation between clinical experts and computer scientists is necessary. Furthermore, patients’ interests should be considered at this stage.

Once a relevant clinical problem best solved by means of ML is identified, the second step is algorithm development. In the predominant case of *supervised learning*, this starts with data selection and establishment of ground truth. Obtaining a sufficient amount of high-quality ground truth data, which can be thought of as “gold standard” used for both ML training and evaluation (e.g., segmentation masks of the left ventricle) is a necessary requirement for creating an algorithm. The amount of data needed depends on the complexity of the problem, the ML algorithm used, and the ratio between the finding of interest and the whole dataset. As a rule of thumb and providing an example from the field of object detection, tasks that are easy to solve for a human reader (e.g., detection and segmentation of a healthy lung within a chest CT scan) will require less training data than the detection of subtle, small changes in a whole-body CT scan. The less training data is available, the better the quality of the data should be. Large training data sets allow for some inaccuracies. It is important to keep in mind that training data quality is a limiting factor for an algorithm’s performance. Therefore, the required data quality also depends on the envisaged performance of the algorithm. The decision how to obtain ground truth again requires both clinical and technical expertise. It revolves around questions such as how data can be extracted from hospital information systems, whether or not to use public databases, and how and by how many experts the ground truth should be established. Whenever patient data leaves primary clinical systems, it is of upmost importance to ensure complete de-identification. In radiology, this includes erasing or overwriting all DICOM tags that contain data privacy relevant information. It is highly recommended to double-check the success of this de-identification process by reviewing DICOM metadata before sending image data. An approach that allows for collaborative training of an ML model without exchanging data samples is “Federated learning” [8]: a copy of an algorithm is downloaded and local data used to further improve it. The resulting changes to the model are summarized in an update that is uploaded and merged with the central consensus model. Preferably, ground truth data is stored in interchangeable formats (e.g., the Neuroimaging Informatics Technology Initiative (NIfTI) data format for segmentations) to assure usability for other projects. This is also in accordance with the FAIR guiding principles for scientific data management and stewardship [9]. Unfortunately, so far no standards have been established for ML and most clinically used post-processing software is not capable of exporting segmentation masks, which limits the reusability of data and impedes reproducibility of studies. There are many other types of ground truth labels, comprising labels on the level of a whole dataset (fracture on radiograph: yes/no) and outcome labels for prediction modeling (patient death: yes/no).

In cardiovascular radiology, most algorithms solve segmentation tasks; therefore, segmentation masks are the predominant type of ground truth in this field. However, also tissue characterization (e.g., T1 and T2 mapping, late gadolinium enhancement) is an increasing part of cardiovascular radiology and needs adequate ground truth labels, e.g., histological results from endomyocardial biopsies in myocarditis. Awareness for potential biases introduced by the composition of datasets is important: an algorithm for clinical outcome prediction developed on a training dataset containing 80% males from country A might not work well on data from female patients in country B. In general, the dataset on which an algorithm is developed should reflect the population on which the algorithm is later applied as good as possible. Either one is aware of these limitations or overcomes the challenge by using large, heterogeneous datasets. The gold standard is to evaluate an algorithm’s performance in clinical practice and their influence on clinical workflows. Finally, a suitable ML technique has to be selected (e.g., random forest or deep convolutional neural networks). This task demands the expertise of the ML expert. This, as other steps, also involves ethical considerations, e.g., whether it is legitimate to develop algorithms on data of highly developed countries only (resulting in better performance in these patient collectives). For a detailed discussion of ethical implications of the use of ML in radiology, we refer to a recently published multisociety statement [10].

The third step is performance evaluation. There is a wide range of statistical tests that can be used, beginning with simpler concepts like sensitivity and specificity for detection tasks to more complex evaluations like the Dice similarity score that ranges from 0 to 1 and quantifies the overlap of two regions of interest [11]. The evaluation method should be defined in advance to avoid method selection bias and involvement of a statistician is highly recommended. It is the responsibility of the radiologist to make sure the selected evaluation method reflects the clinically relevant endpoint. This demands functionality to visually check the validity of the data. Furthermore, the evaluation has to reflect the intended clinical use in the specific patient population the algorithm was designed for. It is also important to consider multicenter testing on different scanner models and patient populations should the algorithm later be used at other clinical centers and in different patient populations.

Finally, if assessed as an effective solution to the clinical problem, translation into clinical practice follows. This last step is at least as challenging as all previous steps and requires expertise in fields that are rarely covered by medical and ML experts, namely in user interface design, graphic design, regulatory matters, and in assuring compatibility with existing hospital IT environments that are subject to changes over time and location. While the creation of a dedicated software package is the most common option, the gold standard is the direct integration of algorithm and its output into existing systems, preferably into Radiology Information Systems/Picture Archiving and Communication (RIS/PACS) systems and radiology reports. This results in a smooth workflow, thereby ensuring acceptance and engagement by users.

**Fig. 2:**
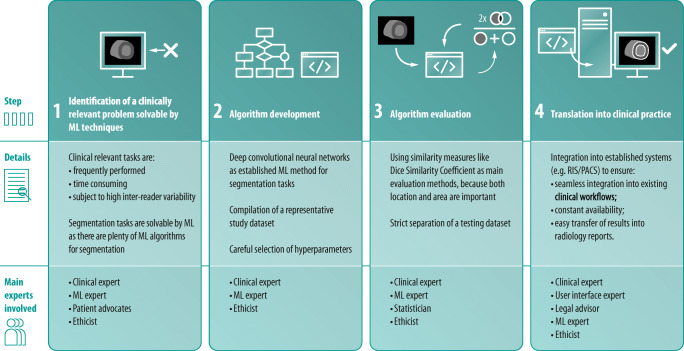
Expertise needed during ML algorithm development and implementation, illustrated with the example of a segmentation task

In our experience, the best results come from a close cooperation between experts from different disciplines. Figure 2 summarizes the four-step process.

(b) Hardware and software requirements

- Consensus statement
*•While some less computationally intensive ML applications can be run on central processing units (CPUs), most currently applied ML algorithms require hardware with dedicated graphics processing units (GPUs).*
*•Experts involved in ML development should make use of online resources for creating, sharing, and discussing ML algorithms.*

Besides human expertise, ML projects have requirements with regard to hardware and software.

### Hardware

Standard CPUs are sufficient to run most non-DL ML algorithms and even DL approaches like deep convolutional neural networks (DCNNs) with few layers. However, DCNNs with multiple layers, which constitute the majority of currently developed ML algorithms of interest for cardiovascular imaging, are more computationally intensive. These algorithms need dedicated hardware with GPUs. Commercially available consumer GPUs with 8 GB or more system memory currently suffice for many applications. A detailed overview on hardware for ML, its performance, and pricing is provided by Tim Dettmers [12]. Alternatively, data can be processed using off-site cloud solutions such as Amazon Web Services.

### Software

**Fig. 3:**
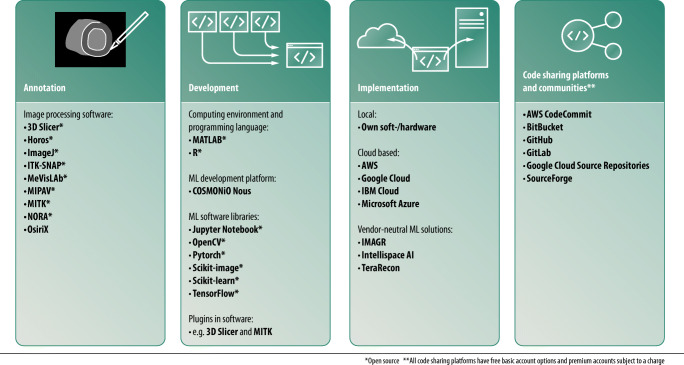
Useful software at different stages of a ML project in radiology

The ML community is fully digital and publishes mostly open source. Practically all relevant resources like software libraries and discussion forums are freely accessible. Jupyter Notebook is a commonly chosen web-based platform to compile ML algorithms (jupyter.org). The platform allows the use of multiple programming languages, including Python, which currently is the most prevalent language in the field of ML (python.org). A programming language can be thought of as the vocabulary and rule system that is used to instruct a computer to perform tasks. ML algorithms can be developed using software libraries like TensorFlow (tensorflow.org), scikit-learn (scikit-learn.org), and PyTorch (pytorch.org). These libraries contain pre-written code and procedures that enable easier and faster software code development. Other alternatives are MATLAB (mathworks.com) and R (www.r-project.org). Once the code is created, it should preferably be shared publicly. GitHub is a common online Git repository for sharing and discussing software code with version control function that allows to retrace a project’s source code history (github.com). Furthermore, anonymization tools are important for ML projects in radiology, because sensitive patient information is part of the DICOM header of each image and data exchange is needed to build large databases with studies from multiple centers. Fortunately, there are numerous free stand-alone tools with batch processing function for Mac OS (e.g., dicomanonymizer.com) and Windows (e.g., rubomedical.com/dicom_anonymizer). The RSNA’s Clinical Trials Processor (CTP) is open-source software that covers the whole image transfer pipeline between data acquisition sites and a principal investigator site with build-in anonymization capability (mirc.rsna.org). Figure 3 provides an overview of useful software and online resources.

## Recommendations regarding study design and reporting

- Consensus statement
*•Based on existing study quality standard frameworks such as SPIRIT and STARD, we propose a list of quality criteria for ML studies in radiology.*

ML studies should be held to the same quality standards as any other diagnostic or prognostic study. Several frameworks exist that define standard protocol items for clinical trials as well as for reporting the results of diagnostic and prognostic studies. Clinical trial protocols should conform to the Standard Protocol Items: Recommendations for Interventional Trials (SPIRIT) checklist [13]. Diagnostic accuracy studies to the Standards for Reporting of Diagnostic Accuracy Studies (STARD) requirements and, at a minimum, should report essential items listed in the 2015 version of the STARD checklist [14]. For prognostic studies, the Transparent Reporting of Multivariable Prediction Model for Individual Prognosis or Diagnosis (TRIPOD) guideline and checklist [15] should be followed. Although these guidelines were not designed with ML studies in mind, they do form a solid basis for providing the details of a ML study in a protocol (SPIRIT), and for reporting results of studies in which ML has been applied (STARD and TRIPOD). Because these guidelines have not been taken up widely in the ML community, efforts are underway to develop ML-specific versions of each of these frameworks. In the meanwhile, we attempt to provide guidance by offering a checklist of items for researchers designing ML studies and for readers assessing the quality of published reports. Our efforts expand upon the recently published editorial by Bluemke et al, which also addresses this topic [16].

### Recommended items for designing and reporting ML studies

In the following section, we provide a list of important considerations when designing and reading studies that employ ML. We have summarized these considerations in a checklist (Table 1) and apply them to a research article that aimed to design a DL algorithm for automatic cardiac chamber segmentation and quantification of left ventricular ejection fraction (LVEF; [17]; Table 2).

1. Which clinical problem is being solved?

**Table 1:** Checklist of items to include when reporting ML studies

1. Which clinical problem is being solved?	
□ Which patients or disease does the study concern?	
□ How can ML improve upon existing diagnostic or prognostic approaches?	
□ What stage of diagnostic pathway is investigated?	
2. Choice of ML model	
□ Which ML model is used?	
□ Which measures are taken to avoid overfitting?	
3. Sample size motivation	
□ Is the sample size clearly motivated?	
□ Which considerations were used to prespecify a sample size?	
□ Is there a statistical analysis plan?	
4. Specification of study design and training, validation, and testing datasets	
□ Is the study prospective or retrospective?	
□ What were the inclusion and exclusion criteria?	
□ How many patients were included for training, validation, and testing?	
□ Was the test dataset kept separate from the training and validation datasets?	
□ Was an external dataset used for validation?*	
□ Who performed external validation?	
5. Standard of reference	
□ What was the standard of reference?	
□ Were existing labels used, or were labels newly created for the study?	
□ How many observers contributed to the standard of reference?	
□ Were observers blinded to the output of the ML algorithm and to labels of other observers?	
6. Reporting of results	
□ Which measures are used to report diagnostic or prognostic accuracy?	
□ Which other measures are used to express agreement between the ML algorithm and the standard of reference?	
□ Are contingency tables given?	
□ Are confidence estimates given?	
7. Are the results explainable?	
□ Is it clear how the ML algorithm came to a specific classification or recommendation?	
□ Which strategies were used to investigate the algorithm’s internal logic?	
8. Can the results be applied in a clinical setting?	
□ Is the dataset representative of the clinical setting in which the model will be applied?	
□ What are significant sources of bias?	
□ For which patients can it be used clinically?	
□ Can the results be implemented at the point of care?	
9. Is the performance reproducible and generalizable?	
□ Has reproducibility been studied?	
□ Has the ML algorithm been validated externally?	
□ Which sources of variation have been studied?	
10. Is there any evidence that the model has an effect on patient outcomes?	
□ Has an effect on patient outcomes been demonstrated?	
11. Is the code available?	
□ Is the software code available? Where is it stored?	
□ Is the fully trained ML model available or should the algorithm be retrained with new data?	
□ Is there a mechanism to study the algorithms’ results over time?	

*Data from another institute or hospital

**Table 2:** Example of applying the checklist to the research article “Automated cardiovascular magnetic resonance imaging analysis with fully convolutional networks” by Bai et al [17]

*1. Which clinical problem is being solved?*	
The study by Bai et al is focused on the fully automated determination of left ventricular ejection fraction (LVEF) and right ventricular ejection fraction (RVEF) on cardiovascular magnetic resonance imaging data, without the need for contour drawing by human experts. Application of ML can reduce the time and human burden of LVEF determination, which can easily take 10–15 min per subject.	
*2. Choice of ML model*	
The investigators used a DL approach with a fully convolutional neural network architecture consisting of 16 layers. Separate networks were trained for three commonly acquired anatomical orientations (short axis as well as vertical and horizontal long axes). Detailed parameters regarding network training are provided. The most important measure to look for with regard to overfitting is the use of strictly separate or “hold out” test dataset (see item 4 below). There was no explicit mention of any other measures taken to avoid overfitting.	
*3. Sample size motivation*	
The DL algorithm was developed using a convenience sample of 4875 subjects participating in the UK Biobank study. No formal sample size calculation was provided. A clear statistical analysis plan is provided in the materials and methods section of the paper. The algorithm was subsequently applied in a study comparing LVEF in normal versus obese subjects. Each of these groups consisted of further 867 patients, also selected from the UK Biobank study. Also for this second study, no formal sample size calculation was provided.	
*4. Specification of study design and training, validation, and testing datasets*	
A random sample of the British population participating in the UK Biobank study was used. As such, this is a retrospective cross-sectional analysis focused on understanding variations in the LVEF in the general population. Detailed inclusion and exclusion criteria were provided. The number of patients used for training, validation, and testing was 3975, 300, and 600 for the short axis segmentation algorithm; 3823, 300, and 600 for the vertical long axis algorithm; and 3782, 300, and 600 for the horizontal long axis algorithm. The investigators do not explicitly mention whether the test dataset was kept separate from the development and validation datasets. The developed algorithms were tested in 1734 additional UK Biobank participants. No external validation outside the UK Biobank was performed.	
*5. Standard of reference*	
The standard of reference consisted of the manual annotations of endocardial and epicardial contours in three anatomical orientations by 8 separate expert annotators. Their level of training and experience is not explicitly mentioned. The investigators also do not mention the number of cases annotated by each individual annotator. Three principal investigators oversaw the annotators, although the investigators do not explicitly mention what exactly their role was. Annotators were blinded for output of the machine learning algorithms.	
*6. Reporting of results*	
To assess the accuracy of the algorithms’ segmentations, the Dice metric, Hausdorff distance, and mean contour distance were calculated, using manual annotations as the standard of reference. In addition, the automatically generated LVEF, right ventricular ejection fraction (RVEF), and the underlying end diastolic and end-systolic volumes of the left and right ventricles and left ventricular (LV) myocardial mass were compared to the reference standard.	
*7. Are the results explainable?*	
The ML algorithms’ outputs can be visually assessed when overlaid on the obtained cardiac MR images, so the end result is easy to verify by human experts. There was no mention of any experiments to investigate the algorithms’ internal logic. However, the DL architecture used in this study has been extensively described by others.	
*8. Can the results be applied in a clinical setting?*	
Because this study concerns a random sample of the British population, the reported results only apply to this group of subjects. The investigators did not test the algorithm in a hospital setting. Based on this study, its accuracy in patients with suspected or known cardiovascular disease is unknown. Nevertheless, the algorithm is capable of running in the hospital on relatively standard computer hardware in combination with a GPU.	
*9. Is the performance reproducible and generalizable?*	
Because the UK Biobank contains cardiac MR images from multiple different scanners and sites, this study provides strong evidence of the generalizability of the algorithms’ performance across different MR hardware platforms and MR scanner operators. However, a standardized image acquisition protocol was used, which does not necessarily correspond to routine clinical practice. Because the algorithm was not tested on non-UK Biobank cardiac MR images, we do not know its performance outside of this domain. Human expert interobserver variation was assessed by comparing contours drawn three expert observers. Finally, the automatically generated contours for 250 randomly selected test subjects were visually assessed by two experienced image analysts.	
*10. Is there any evidence that the model has an effect on patient outcomes?*	
The investigators focused on development of an algorithm for automated ventricular ejection fraction measurement. Outcome was not studied.	
*11. Is the code available?*	
The cardiac MR data including the segmentations are available upon request for health-related research in the public interest. The software code is available on GitHub. It is unclear if the algorithm needs to be retrained with new data.	

A clear description of the clinical problem and rationale for the study should be provided, taking into account existing approaches and how they fall short. This includes the specification of the disease in question and a clear description of the subjects or patients studied. It is also important to hypothesize how ML approaches may improve upon existing approaches such as conventional statistical approaches to solve the problem. Other relevant questions include the stage of the disease in question and place in the diagnostic pathway.

2. Choice of ML model

The choice of ML model should be clearly motivated since there is a wide variety of approaches, which may result in different results. It is also important to explicitly discuss overfitting and approaches used to mitigate this problem. Overfitting occurs when ML models are trained to predict training data too well, which results in the inability to generalize to new, unseen data. An overview of commonly used ML models and their characteristics as well as approaches that can be used to deal with overfitting is provided by Liu et al in their review article [18]. Technical details of the algorithm including hyperparameters should be specified to foster transparency and replicability.

3. Sample size motivation

In contrast to the recommendations made in the STARD and CONSORT guidelines, most ML studies have not explicitly considered sample size when designing the study and are often based on convenience samples. However, sample size and a statistical analysis plan should ideally be prespecified. Although there are presently no clear guidelines on how to calculate a sample size in ML studies, the number of subjects or datasets can be prespecified according to considerations such as the minimal clinical difference of interest or the expectation that ML is able to generate equivalent results to human observers on a certain task. Furthermore, sample sizes used by other researchers to solve comparable problems might be a good indicator.

4. Specification of study design and training, validation, and testing datasets

Algorithm development demands data for training, validation, and testing. Investigators should specify how the data was split into each of these categories. It is of utmost importance to strictly separate the testing dataset from the other datasets to obtain a realistic estimate of model performance. This is also a requirement for regulatory approval of ML-based computer-assisted detection devices from the United States Food and Drug Administration (FDA) [19]. Ideally, validation is performed not only on internal data (from the same department or institute) but also on an external dataset by independent researchers.

5. Standard of reference

A key consideration in ML studies is selection and quality of the reference standard or ground truth. Researchers should precisely specify how and by whom ground truth data were labeled, including the level of experience of each observer. It is important to take into account interobserver variability between experts and to describe how disagreements are resolved (e.g., by demanding that observers reach a consensus, or by adjudicating any differences by a separate observer). It should be noted whether existing labels were used (e.g., from radiology reports or electronic health records), or new labels were created. Finally, experts labeling the data should ideally work independently from each other because this will facilitate measurement of interobserver agreement between human experts.

6. Reporting of results

Analogous to conventional diagnostic studies, contingency tables with the number of true positive, true negative, false positive, and false-negative classifications should be given at the prespecified chosen classifier threshold. Other useful measures include the area under the receiver operating curve (AUC) and Bland-Altman plots [20]. It is important to note that terminology in ML studies may be different from the terminology used in the medical literature. Sensitivity is equivalent to “recall” and “precision” denotes positive predictive value. The F1 score is a compound measure of precision and recall and its use is therefore highly recommended. Table 3 summarizes measures frequently used in ML. Confidence intervals should be reported for all of these measures. In image segmentation and analysis tasks, measures of how well the ML algorithm performs compared to the standard of reference should be given. These typically include the Dice coefficient (a measure of how well the ML generated contours overlap with the standard of reference contours), the mean contour distance (the mean distance between two segmentation contours), and the Hausdorff distance (the maximum distance between the 2 segmentation contours) [11].

7. Are the results explainable?

**Table 3:** Performance metrics frequently used in ML

Metric	Definition and details
Recall	Fraction of true positive (TP) instances among the instances predicted to be positive by an algorithm, including false positive (FP) instances (synonym for “positive predictive value”) Recall = $\frac{TP}{TP+ FP}$
Precision	Fraction of the instances predicted to be positive by an algorithm among all TP instances, including false negative (FN) instances (synonym for “sensitivity”) Precision = $\frac{TP}{TP+ FN}$
Accuracy	Fraction of TP and true negatively (TN) predicted instances among all instances. Accuracy = $\frac{TP+ TN}{TP+ TN+ FP+ FN}$
F_1_-score	Harmonic mean of precision and recall. Ranges from 0 to 1 (meaning perfect precision and recall). Important measure, because both high precision and recall are needed for high F_1_ scores. F_1_ = 2 * ($\frac{\mathrm{precision}\ast \mathrm{recall}}{\mathrm{precision}+\mathrm{recall}}$)
False-positive findings	Negative instances falsely predicted to be positive by an algorithm. Numbers of false-positive findings are very important in ML, because too many of them render algorithms useless. Investigating the reasons for false-positive findings may help to develop strategies to avoid them, but requires domain knowledge in the field of application.
ROC curve	Receiver operating characteristic curve. Graph illustrating the discriminative ability of a classifier. Sensitivity (*Y*-axis) plotted against the false-positive rate (*X*-axis) for different classification thresholds. The area under the curve (AUC) measures the 2D area underneath the ROC curve and provides an aggregate measure of performance.
Intersection-Over-Union (IoU)	Important measure to assess the performance of algorithms for segmentation tasks. Overlap between two regions of interest, mostly of a ground truth segmentation and a predicted segmentation, e.g., of the left ventricle. Ranges from 0 to 1, with 1 indicating perfect overlap. IoU = $\frac{\mathrm{Area}\ \mathrm{of}\ \mathrm{overlap}}{\mathrm{Area}\ \mathrm{of}\ \mathrm{union}}$.
Dice similarity coefficient (DSC)	Another important measure in assessing segmentation algorithms. Ranges from 0 to 1, with 1 indicating perfect overlap. DSC = $\frac{2\ast \mathrm{Area}\ \mathrm{of}\ \mathrm{overlap}}{\mathrm{Total}\kern0.5em \mathrm{area}\ \mathrm{of}\ \mathrm{objects}}$

Because of the large number of parameters involved, interpreting the results of ML studies can be challenging, especially when working with DL algorithms. This consideration is particularly pertinent when important treatment decisions are contingent upon the results generated by the algorithm. Saliency mapping enables the identification of morphological features in the input image underlying the model’s prediction and can help to investigate the algorithm’s internal logic. Visual feedback about the model’s predictions is very important to understand whether networks learn patterns agreeing with accepted pathophysiological features or biologically unknown, potentially irrelevant features.

8. Can the results be applied in a clinical setting?

Machine learning studies designed to solve a specific clinical problem should explicitly consider whether the results apply to a real-world clinical setting. This includes discussion of how representative the dataset used for derivation and testing of the model is of the clinical setting in which it will be applied. Any sources of bias, in particular class imbalance and spectrum bias, should be identified and discussed. Considering these factors can enable more precise identification of patients in which the algorithm can be used clinically, or in which groups of patients and clinical scenarios additional validation is needed. Investigators should also consider if and how the algorithm can be used at the point of care, including issues like availability of the algorithm (e.g., on-premise or via cloud solutions), how fast results are available (e.g., in real-time or with a delay), and how results are visualized in order to check the model’s predictions.

9. Is performance reproducible and generalizable?

To date, in most reports on ML, model development, tuning, and testing have been performed on a convenience sample of locally available data. Although many of these reports have demonstrated encouraging results, it is important to investigate the reproducibility of the model and to perform an external validation, preferably on multiple datasets from other independent institutes and investigators. External validation is important to investigate the robustness of the model to e.g. differences in image acquisition and reconstruction methods between different vendors and institutes and differences in referral patterns and variability in the prevalence of the condition of interest. Conversely, we also believe it is advisable to validate external algorithms prior to local use, especially if the algorithms` results are used for automated analysis with results directly transferred into clinical reports instead of use as a second reading tool.

10. Is there any evidence that the model has an effect on patient outcomes?

Although one of the first proofs of concept in the development of an ML algorithm is the investigation of its diagnostic accuracy, investigators and readers should ask themselves the question whether there is any evidence of an effect on patient outcomes. This is especially important for algorithms used for treatment recommendations and detection of unrequested findings. Ideally, this should be investigated in prospective, randomized clinical trials, as is the case for conventional interventions. These considerations also help to detect and mitigate reasons for missing impact of diagnostically well performing algorithms on patient outcomes, such as suboptimal communication of results.

11. Is the code available?

Transparency regarding an ML model’s design and function is key to clinical acceptance. Making the computer code available to other investigators is a major step towards this goal and is increasingly becoming a condition for obtaining funding as well as acceptance of studies in high-quality, peer-reviewed journals. The GitHub platform facilitates free and rapid dissemination of software code with basic quality checks. Investigators should state whether the source code of their algorithm will be made available and under which conditions. If not, specific reasons should be given. Making the software code available enables other researchers to independently investigate whether reported results can be reproduced and to improve model performance. Furthermore, it enables the evaluation of a model’s performance over a prolonged period of time.

## Insights of a systematic literature review on applications of ML in cardiac radiology

**Fig. 4:**
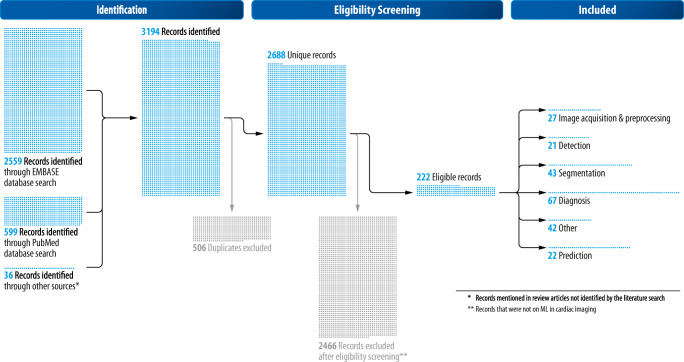
Search and review flow diagram

To identify articles on the application of ML in cardiac radiology, a comprehensive search for articles in PubMed and EMBASE databases was conducted. The search identified all articles in the English language registered no later than 31.01.2020 (*n* = 599 in PubMed; *n* = 2559 in EMBASE). Supplement 1 documents the search strings. Figure 4 displays the exact search and review workflow that included the removal of duplicates (*n* = 506) with the auto-function of the literature management software (Mendeley) and the exclusion of articles that were not on ML in cardiac radiology by manual screening (*n* = 2466). In the next step, the remaining relevant articles (*n* = 222) were classified into five categories according to the function of the ML applications: (a) image acquisition and preprocessing, (b) detection, (c) segmentation, (d) diagnosis, (e) prediction, and (f) other. The relation of those categories is sequential; e.g., detection is a prerequisite for segmentation. The studies were attributed to the most advanced category according to the purpose of the given algorithm.

**Fig. 5:**
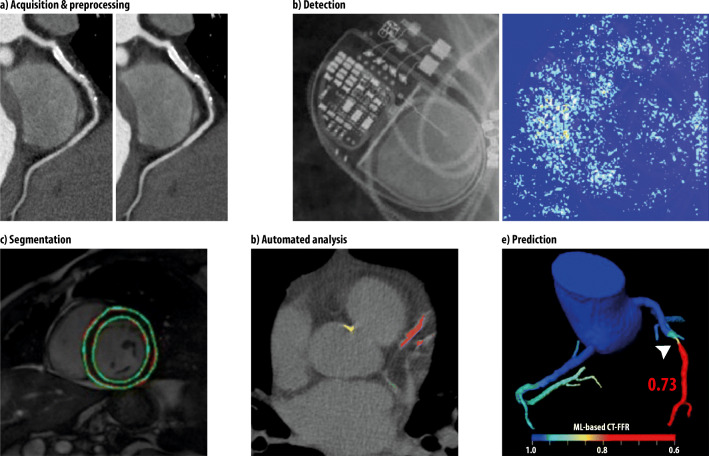
Examples of application of ML in cardiac radiology. **a** Curved multiplanar reformation of CTAs with multiple plaques and a stent in the right coronary artery; standard hybrid iterative image reconstruction on the left, image processed with an ML algorithm with reduced noise on the right [21]. **b** Correctly identified Advisa device on a plain radiograph (left) with the according saliency map (right) that visualizes the neural networks attention [22]. **c** Segmentation of the LV on MRI by a DCNN with automatically detected contours in green color [23]. **d** Automated detection and quantification of calcifications on non-contrast CT scans (red: left anterior descending coronary artery; green: left circumflex coronary artery; yellow: thoracic aorta) [24]. **e** ML-based CT fractional flow reserve predicting obstructive stenosis in the mid left anterior descending coronary artery [25]

At this point, we briefly mention an example per category; Fig. 5 presents corresponding images: (a) Tatsugami et al used a DCNN with 10 layers to reduce the image noise of CT angiography images. The mean image noise was significantly lower than that of images reconstructed with standard hybrid iterative reconstruction alone (18.5 ± 2.8 HU vs. 23.0 ± 4.6 HU) [21]. (b) Howard et al developed five neural networks on 1676 images to detect and identify cardiac pacemakers and defibrillators on chest radiographs. They report an accuracy of 99.6% and even classified specific model groups of the devices [22]. (c) Romaguera et al used a DCNN to segment the left ventricle in short-axis cardiac MRI images and found a Dice score of 0.92, a sensitivity of 0.92, and a specificity of 1.00 [23]. (d) Lessmann et al developed and tested DCNNs for an automated calcium scoring on 1744 non-ECG-gated CT scans without contrast. They report an F1 score of 0.89 for calcium scoring of coronaries on soft kernel reconstructions [24]. (e) Coenen et al used a neural network with four layers to predict the hemodynamic relevance of coronary artery stenoses from CTA data alone by using the ML-based FFR (fractional flow reserve) with invasively measured FFR as a standard of reference. They report an improved diagnostic accuracy of CTA-based assessment of stenosis from 71 to 85% (sensitivity: 89%; specificity 76%) [25].

**Fig. 6:**
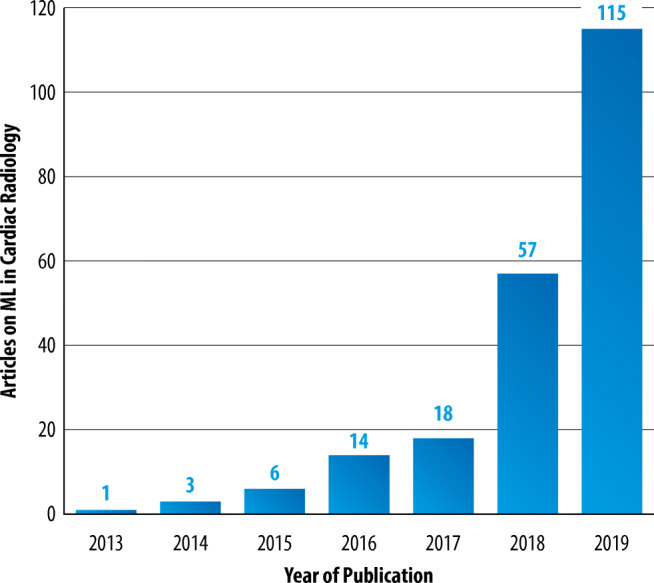
Number of articles on ML in cardiac radiology (*Y*-axis) published per year between 2013 and 2019 (*X*-axis), as resulting from the structured literature review. Studies published earlier than 2013 and in 01/2020 are not included for reasons of clarity

**Fig. 7:**
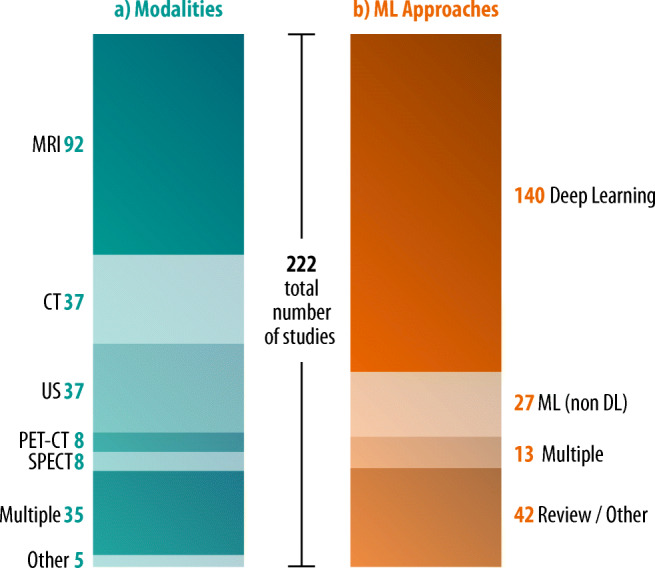
**a** Modalities involved and (**b**) types of ML approaches used in studies on ML in cardiac radiology (*n* = 222)

Figure 6 demonstrates the exponentially increasing number of publications on ML in cardiac radiology since 2013. Figure 7 shows the distribution of modalities and the ML techniques that were covered in the research articles, with MRI being the predominant modality (41.4%) and DL being the most frequently used ML technique (63.1%).

Supplement 2 provides a detailed literature review on ML in cardiac radiology highlighting and discussing important studies in all categories. Supplement 3 contains the complete reference list of all studies resulting from the literature search and a table with detailed information on the studies.

## ML in cardiovascular radiology: opportunities and challenges

- Opportunities

- Consensus statement
*•ML algorithms provide opportunities along the whole task-pipeline of cardiovascular radiology.*

Machine learning offers great opportunities in cardiovascular imaging from the perspective of multiple stakeholders. First and foremost, from a patient perspective, there are opportunities to avoid unnecessary imaging. Should imaging be deemed necessary, ML offers opportunities to do so with shorter imaging protocols and lower radiation doses. Once the images are acquired, automated post-processing facilitated by ML offers quicker results compared to current workflows and could reduce interobserver variation and aid in prioritizing studies with urgent findings. ML can also be used to extract additional relevant information from images. Some examples relevant to cardiovascular imaging include extraction of volumes of *all* cardiac chambers instead of just the LV, more detailed analysis of cardiac motion patterns, and quantification of the amount of pericoronary and pericardial fat as well as the amount of liver fat. When reliable algorithms capable of assigning a diagnosis become available, this could reduce diagnostic error by serving as a “second reader.” Finally, ML can aid in automatic extraction of unrequested but prognostically relevant information. For example, automated detection or exclusion of pulmonary nodules or abnormalities in other organ systems depicted in the field-of-view would be useful for radiologists specialized in cardiovascular imaging. Furthermore, ML algorithms can be used to create detailed local, national, and international databases with normal values for clinical comparison. This will also enable the detection of smaller effect sizes and more precise results.

(b) Challenges

- Consensus statement
*•ML algorithms were initially developed to solve problems in non-medical domains. Due to peculiarities of health-related data like high inter-reader variability, dispersed data storage, and data privacy issues, ML projects in radiology are facing specific challenges.*
*•The medical research community should strive for the compilation of multicenter datasets that are currently lacking in the field of cardiovascular imaging.*
*•Further challenges encompass rare disease and/or anatomical variants and compliance with legal frameworks.*

The compilation of high-quality datasets for ML projects in radiology is hampered by some peculiarities of health-related data. First, there is the issue of significant inter- and intrareader variability fostered by the fact that many categories in medicine are not as distinct as those of everyday objects such as dogs or cars. Second is the complexity of medical image interpretation. For example, a small hyperintense streak in late gadolinium enhancement imaging may be a hyperintensity artifact or a true scar. For clarification, one needs to integrate additional information such as whether there is an implanted cardiac device or not. Third, there is a lack of standardization in the acquisition of medical data. In radiology, heterogeneity is introduced by differing vendors of hardware, software, and unstandardized acquisition parameters. This is true for imaging data, and also for other diagnostic tests and therefore prevents “one-fits-all” solutions. The fourth challenge concerns the non-standardized format and dispersed nature of health data. While in other areas like engineering data is registered in interchangeable systems often designed from scratch, data in hospitals are mostly stored in dispersed, historically grown data silos in multiple data formats. The fifth challenge, especially relevant to cardiovascular radiology, is dealing with higher dimensional imaging data. For example, cardiac cine images are four-dimensional, while ML algorithms are traditionally designed to cope with data in two dimensions. Solutions to this challenge are either complex, or reduce information (processing of a 3D CT dataset as a series of multiple 2D images). Another important challenge to ML projects in radiology is strict standards of data privacy.

All this makes the creation of high-quality ground truth datasets in healthcare challenging and expensive. As a result, datasets in healthcare-related ML projects tend to be much smaller compared to the non-medical domain: the famous contest for everyday object detection, the ImageNet challenge, encompasses over 14 million images (http://www.image-net.org/about-stats), while ML studies in cardiovascular radiology often comprise less than 100 cases. Public datasets like the ChestX-ray8 dataset provided by the NIH containing more than 100,000 frontal-view radiographs with eight disease labels are important initiatives to overcome this problem. However, labels need critical quality review, which requires medical domain knowledge. For cardiovascular radiology, comparable datasets are currently lacking and professional societies can play an important role in the assembly of large publicly available datasets with high-quality ground truth labels to allow for an objective comparison of different ML algorithms. Registries like the ESCR MR/CT registry (https://www.mrct-registry.org), providing large standardized data sets for further analyses with currently > 300,000 de-identified examinations [26], are also an important contribution in this direction.

Apart from image data–related problems, there are other challenges. First, there is the problem of rare disease entities. ML algorithms need a sufficient amount of training examples to detect patterns, ideally including examples at the extreme ends of the disease spectrum. However, many radiologic disease patterns are rarely seen, like congenital heart diseases, and ensuring a fully representative training dataset remains a difficulty. Second, legal issues: Machine learning algorithms, although highly accurate for many tasks, are never perfect and discussions on legal liability for incorrect or missed diagnoses are ongoing [27]. Third, the question of acceptance: will physicians and patients be willing to trust judgments of algorithms that are a “black box” to them? However, trust in systems not fully understandable to us is part of day-to-day life.

## Conclusion

The number of scientific studies published on ML in cardiovascular imaging has been exponentially growing with more than 100 research articles in 2019. The majority concerned MRI studies using DCNNs for image segmentation tasks, but ML algorithms can also help to shorten imaging protocols and extract more information from the same imaging data. The prerequisites for ML to make important contributions in the field of radiology are now in place: freely available open-source software, vast amounts of digital radiology data in most countries, and an increasing presence of well-trained experts to train and clinically supervise ML. Furthermore, online transfer of data and ML models has become convenient.

However, to accomplish this enormous potential, the field of radiology needs to develop common quality standards regarding ML applications and studies. We highlight the need for a detailed description of datasets and methodology used. Furthermore, in the course of ML algorithm development that aims at having a clinical impact, cooperation of professionals from multiple backgrounds is required.

## Electronic supplementary material

ESM 1 (DOCX 89 kb)

## Abbreviations

AI: Artificial intelligence
ASCI: Asian Society of Cardiac Imaging
AUC: Area under the curve
CONSORT: Consolidated Standards of Reporting Trials
CPU: Central processing unit
CT: Computed tomography
DCNN: Deep convolutional neural network
DL: Deep learning
DSC: Dice similarity coefficient
ECG: Electrocardiography
ESCR: European Society of Cardiovascular Radiology
EUSOMII: European Society of Medical Imaging Informatics
FDA: Food and Drug Administration
FFR: Fractional flow reserve
FN: False negative
FP: False positive
GPU: Graphics processing unit
HU: Hounsfield unit
IoU: Intersection-Over-Union
LV: Left ventricular/left ventricle
LVEF: Left ventricular ejection fraction
ML: Machine learning
MRI: Magnetic resonance imaging
NASCI: North American Society for Cardiovascular Imaging
NIfTI: Neuroimaging Informatics Technology Initiative
PACS: Picture archiving and communication system
RIS: Radiology information system
RVEF: Right ventricular ejection fraction
SCMR: Society of Cardiovascular Magnetic Resonance
SPIRIT: Standard Protocol Items: Recommendations for Interventional Trials
SSCT: Society of Cardiovascular Computed Tomography
STARD: Standards for Reporting of Diagnostic Accuracy Studies
TN: True negative
TP: True positive
TRIPOD: Transparent Reporting of Multivariable Prediction Model for Individual Prognosis or Diagnosis
US: Ultrasound
