# Supplementary material for: Machine learning in cardiovascular radiology: ESCR position statement on design requirements, quality assessment, current applications, opportunities, and challenges
Source: Eur Radiol. 2020 Nov 19;31(6):3909–22. doi: 10.1007/s00330-020-07417-0 (PMC8128798; doi:10.1007/s00330-020-07417-0)
Supplement: Supplementary file 1 — (DOCX 89 kb) [file 330_2020_7417_MOESM1_ESM.docx]

**Supplements**

Supplement 1: Search strings used during systematic literature search.

Supplement 2: Discussion of current literature on applications of ML in cardiac imaging:

- 1. Image acquisition & preprocessing
  2. Detection
  3. Segmentation
  4. Diagnosis
  5. Prognosis
  6. Other

Supplement 3: Table with detailed information on the publications resulting from the literature search and complete list of references.

**Supplement 1: Search strings used for systematic literature search.**

- **PubMed:** "cardiac" [tiab] AND ("AI" [tiab] OR "artificial intelligence" [tiab] OR "machine learning" [tiab] OR "DL" [tiab] OR "deep learning" [tiab] OR "DCNN" [tiab] OR "deep convolutional" [tiab]) AND (radiology [tiab] OR imaging [tiab] OR CT [tiab] OR MRI [tiab] OR MR [tiab] OR ultrasound [tiab] OR radiograph [tiab])
- **EMBASE:** 'cardiac':ti,ab,kw AND (ai:ti,ab,kw OR 'artificial intelligence':ti,ab,kw OR 'machine learning':ti,ab,kw OR 'dl':ti,ab,kw OR 'deep learning':ti,ab,kw OR 'dcnn':ti,ab,kw OR 'deep convolutional':ti,ab,kw) AND (radiology:ti,ab,kw OR imaging:ti,ab,kw OR ct:ti,ab,kw OR mri:ti,ab,kw OR mr:ti,ab,kw OR ultrasound:ti,ab,kw OR radiograph:ti,ab,kw)

**Date of search:** 31.01.2020

**Supplement 2: Discussion of current literature on applications of ML in cardiac imaging**

**a) Image Acquisition & preprocessing**

A problem in Cardiac Imaging often tackled by means of ML is that of motion artifacts [1–5]. In detail, Lossau and colleagues trained a patch-based model for the detection of motion artifacts on ECG-triggered coronary CT angiography. Original CT images were split into multiple subsamples (patches) that were consequently classified individually, a frequently applied method to transfer 3D data into a format easily processable for DL algorithms. They report an accuracy of up to 93.3% (SD: 1.8%), however they tested on data from eight cardiac CTs from one center only [2]. A possible area of application of such algorithms for motion artifact detection is real-time monitoring of imaging quality. Küstner et al. went one step further and demonstrated the feasibility of several DL frameworks based on variation auto encoders (VAEs) and generative adversarial networks (GANs) for the retrospective correction of different kinds of motion artifacts on MR images [3]. Tatsugami developed a DL-based image restoration algorithm that could significantly improve the signal-to-noise ratio of coronary CT angiography [6].

Long acquisition times are a main challenge of cardiac MRI. Two groups demonstrated superiority of ML approaches compared to classical methods of reconstruction of substantially undersampled cardiac MRI data, thereby potentially reducing acquisition time of cardiac MRI protocols [7, 8]. In detail, Qin and colleagues used an end-to-end architecture combining a traditional iterative reconstruction algorithm with a recurrent neural network for the reconstruction of dynamic cardiac MRI images that outperformed other reconstruction methods for in terms of accuracy (similarity indices: 0.99 compared to 0.95-0.98) and speed (3-6 seconds compared to 8-451 seconds processing time) [7]. De Vos et al. proposed a DL framework to align cardiac cine MRI images and reported accuracy comparable to traditional methods, however at a 350 times faster speed of 39 ms [67]. Margeta and colleagues used multiple architectures (random forest, CNNs) to recognize acquisition planes in cardiac MRI (F1 score: 0.98, SD: 0.02) [9].

**b) Detection**

The detection of findings within a scan is a basic ability that is a prerequisite for subsequent automated image analysis. The easier task of detecting anatomical structures and pathology on 2D radiographs seems to be no challenge to present-day algorithms: examples in cardiac imaging are the detection of cardiomegaly [10] or of cardiac rhythm devices on chest radiographs [11] (accuracy: 99.6%; CI: 97.5%-100.0%). This is explained by the fact that the vast majority of ML algorithms for radiology image analysis are modified versions of algorithms initially programmed for the detection of findings on non-medical 2D images (e.g. of cars on photographs).

But even on 3D medical images, detection performance of AI algorithms is reported to be good. Wolterink et al. proposed an algorithm for the detection of centerlines of coronary arteries in cardiac CT angiography and found an almost perfect overlap of proposed and reference centerlines [12]. Emad and colleagues reported an accuracy of 98.66% for the detection of the left ventricle on MRI using a patch-based convolutional neural network (CNN; sensitivity: 83.91%; Specificity 99.07%) [13]. Ostvik and colleagues used a CNN for real-time classification of standard views in transthoracic echocardiography with sensitivities from 93.1% to 99.6% and positive predictive values of 96.2% to 99.4% [14].

**c) Segmentation**

Most segmentation algorithms in Cardiac Radiology aim at delineating the four heart chambers. Algorithms for multiple modalities have been presented: CT [15, 16], MRI [17–30], Echocardiography [31–33] and SPECT [34]. Another area of application is the segmentation of image findings.

CT

Dormer and colleagues developed a patch-based CNN to segment the four chambers on CT and found an average accuracy of 87.2% (SD: 3.3%) for the classification of patches (classes: left ventricle, right ventricle, left atrium, right atrium, background). However, they did report neither sensitivity/specificity nor a method to transfer the classified patches to coherent 2D/3D segmentations. Furthermore, both training and testing was performed on a dataset of only 11 CTs [15]. Lin et al. trained and tested two 3D U-Nets with 160 non-contrast CTs and found high Dice-Scores ranging from 0.82 (SD: 0.07) to 0.96 (SC: 0.02) for the four chambers and whole-heart segmentation [16]. However, given the fact that even small deviations can result in exceeding a clinically relevant threshold, required levels of precision and reproducibility are high.

MRI

Avendi et al. developed a DL-algorithm for segmentation of the left ventricle (LV) on short axes on MRI datasets and reached a DICE score of 96.7% (SD: 5.7), training and testing on 45 MRI datasets [35]. The deep convolutional neural network (DCNN) proposed by Molaei and colleagues for LV-myocardium-segmentation is based on ImageNet, and was developed and tested on 5,011 MRI images from 33 patients. They reported a per-pixel sensitivity of 84.1% and a specificity of 98.4% in predicting whether a pixel belongs to the LV-myocardium or not. While most of the above mentioned projects focused solely on LV-segmentation, Isensee et al. proposed a more comprehensive U-Net-based algorithm that showed good performance for the segmentation of the LV (Dice score: 0.95), right ventricle (Dice score: 0.92) and the LV myocardium (Dice score: 0.91) on an independent test set and capable of processing the entire cardiac cycle [21]. Du et al. reported similar performance measures for the three anatomical areas using an encoder-decoder approach [19].

Ultrasound

Arafati and colleagues described a fully-connected DCNN to automatically annotate heart chamber boundaries on echocardiographic images. They trained on imaging data of 100 healthy subjects and report a Dice score of 89.6% (left ventricle) and 85.4% (right ventricle) [32]. Leclerc et al. developed and tested multiple encoder-decoder architectures to segment end-diastolic and end-systolic left ventricular volumes [33]. The best performing approach was a U-Net with Dice coefficients between 0.92 and 0.95, indicating very good segmentation accuracy. However, they excluded datasets with bad image quality from the test set.

SPECT

In a feasibility study based on imaging data of 56 patients, Wang et al. proposed an end-to-end CNN to segment the LV myocardium in gated myocardial perfusion SPECT imaging. They found high correlation of derived LV myocardium volumes and ground truth volumes (r = 0.91 +- 0.1) [34].

Segmentation of other cardiac findings

Commandeur et al. developed a CNN for the segmentation and quantification of epicardial adipose tissue and thoracic adipose tissue on non-contrast coronary artery calcium CT. They evaluated the algorithm on a dataset consisting of 250 asymptomatic individuals, and found Dice score coefficients when comparing automated segmentations with ground-truth-segmentations of 0.82 and 0.91, respectively, indicating feasibility of the approach for a fully automated workflow possibly contributing to cardiovascular risk stratification [36]. Fahmy and colleagues used a DCNN to segment myocardium scar tissue on late-gadolinium enhanced MR images in patients with hypertrophic cardiomyopathy to allow for automated scar quantification. Automated segmentation was achieved in 0.26s/image. While they found strong correlation of automatic and manually segmented scar volumes (r = 0.84), the Dice score was only 0.57 +- 0.23, indicating unprecise finding detection. This discrepancy may be explained by the fact that the scars were small in size, which makes Dice scores more sensitive to smaller deviations. Two groups presented algorithms for the automated quantification of myocardial scar tissue on cardiac MRI [37, 38]. In detail, Moccia et al. introduced a CNN algorithm for the segmentation of scar tissue in the LV wall on late gadolinium enhancement images. They report a median DICE similarity coefficient of 71% for a fully automated approach [37].

**d) Diagnosis**

There is increasing literature on frameworks that facilitate an automated analysis of cardiac function on cardiac MRI [39–42]. Bai and colleagues presented a fully CNN that had been trained on a dataset from the UK Biobank, consisting of 4,875 subjects with over 90,000 pixel-wise annotated images. After segmentation on short-axis images, the mean absolute differences between automated measurements and manual ground-truth measurements were as low as 6.1 ml (LV end-diastolic volume), 5.3 ml (LV end-systolic volume), 7 gram (LV mass), 8.5 ml (RV end-diastolic volume) and 7.2 ml (RV end-systolic mass), respectively [39]. Of interest, this performance is comparable to human inter-observer variability. Winther et al. trained an U-net-based algorithm on 253 manually segmented cases and tested on multicenter datasets with > 1000 cases. They reported high intraclass correlation coefficients (ICCs) for LV ejection fraction (80% - 98%), RV ejection fraction (87% - 96%), LV ventricular mass (94%-95%), and RV ventricular mass (83%) [40]. This is a strong indicator that, despite parameter heterogeneity in MR, it is possible to create a generalizable algorithm for this task. Ruijsink et al. presented a framework consisting of two CNNs, the first for an image quality check to reject images with artifacts and the second for full cycle segmentation on short axis, 4- and 2-chamber views. The derived functional values correlated highly with manual analysis (ventricular volumes r > 0.95; filling and ejection rates all ≥ 0.93). However, 11% of all cases were rejected for reasons of image quality [41]. Similarly, Curiale et al. reported correlation coefficients between 0.93 and 0.99 for several physiological measures of the left ventricle (end-systolic and diastolic volumes, myocardial mass, ejection fraction and stroke volume / cardiac output) [42].

Jafari et al. used a CNN to estimate LV ejection fraction based on ultrasound two- and four-chamber views in real-time. The network segments the LV and detects anatomical landmarks. The median absolute error for LVEF estimation was only 6.2%, tested on a dataset of 427 patients [43].

Another important area of research is automatic calcium scoring. Lessmann et al. used a two-stage CNN to detect, quantify and localize (LAD, LCX, RCA) coronary calcifications on low-dose chest CT [44] and found very good performance on soft filter reconstructions (F1-score 0.89). This algorithm is also able to identify calcifications of the thoracic aorta (F1-score 0.89), and – with only low to moderate accuracy – of the aortic valve (F1-score 0.67) and of the mitral valve (F1-score 0.55). They trained and tested on 1,744 non-gated, low-dose chest CT scans contained in the National Lung Screening Trial database.

Takx et al. demonstrated the ability of an algorithm combining traditional image processing techniques like thresholding with a support-vector-machine to automatically determine the calcium score in non-gated, low-dose chest CTs [45]. They found very good reliability for the risk categories of the Agatston scores (K=0.85), albeit the algorithm systematically underestimated calcium score values.

Zreik and colleagues presented a ML-pipeline with CNN, an unsupervised convolutional autoencoder and an SVM classifier to automatically identify patients with functionally significant coronary artery stenosis according to fractional flow reserve determined during invasive coronary angiography, based solely on an analysis of the LV myocardium in CTAs (AUC: 0.74, SD: 0.02; however: sensitivity of only 70% at a specificity of 71%) [46].

**d) Prediction**

Regarding prediction of patient outcome with ML, there are some reports on frameworks that use semi-automatically or manually extracted imaging parameters to predict all-cause mortality of patients with suspected coronary artery disease from coronary CTA and clinical parameters [47] and survival of patients with pulmonary hypertension from 3D right ventricular motion characteristics [161]. Commandeur et al. used combined clinical and imaging information to predict the long-term risk of myocardial infarction and cardiac death [164]. So far, there are no fully automated end-to-end systems for outcome prediction from cardiac imaging data. Coenen et al. used a neural network with four layers to predict the hemodynamical relevance of coronary stenoses from CTA alone with invasively measured fractional flow reserve as standard of reference. They report an improved diagnostic accuracy of CTA-based assessment of stenosis from 71% to 85% (sensitivity: 89%; specificity 76%) [163].

| **Supplement 3: Table with detailed information on the publications resulting from the literature search and complete list of references.** | | | | | | |
| --- | --- | --- | --- | --- | --- | --- |
| **#** | **Authors** | **Year** | **Modality** | **Technique** | **Group** | **Reference** |
| 1 | Bahrami et al. | 2019 | MRI | DL | A&P | [48] |
| 2 | Biswas et al. | 2019 | MRI | DL | A&P | [49] |
| 3 | Blansit et al. | 2019 | MRI | DL | A&P | [50] |
| 4 | Chen et al. | 2019 | MRI | DL | A&P | [51] |
| 5 | Fan et al. | 2020 | MRI | DL | A&P | [52] |
| 6 | Ghodrati et al. | 2019 | MRI | DL | A&P | [53] |
| 7 | Kofler et al. | 2019 | MRI | DL | A&P | [54] |
| 8 | Küstner et al. | 2019 | MRI | DL | A&P | [3] |
| 9 | Margeta et al. | 2017 | MRI | DL | A&P | [9] |
| 10 | Qin et al. | 2019 | MRI | DL | A&P | [7] |
| 11 | Sandino et al. | 2019 | MRI | DL | A&P | [55] |
| 12 | Scannell et al. | 2019 | MRI | DL | A&P | [56] |
| 13 | Schlemper et al. | 2018 | MRI | DL | A&P | [8] |
| 14 | Zhao et al. | 2019 | MRI | DL | A&P | [57] |
| 15 | Zhou et al. | 2019 | MRI | DL | A&P | [58] |
| 16 | Kim et al. | 2019 | CT | DL | A&P | [59] |
| 17 | Lossau et al. | 2019 | CT | DL | A&P | [2] |
| 18 | Lossau et al. | 2019 | CT | DL | A&P | [60] |
| 19 | Lv et al. | 2019 | CT | DL | A&P | [61] |
| 20 | Tatsugami et al. | 2019 | CT | DL | A&P | [6] |
| 21 | Yan et al. | 2016 | CT | DL | A&P | [62] |
| 22 | Nakanishi et al. | 2018 | CT | ML-Non-DL | A&P | [4] |
| 23 | Diller et al. | 2019 | US | DL | A&P | [63] |
| 24 | Fung et al. | 2019 | US | DL | A&P | [64] |
| 25 | Karuzasl et al. | 2019 | US | DL | A&P | [65] |
| 26 | Ladefoged et al. | 2019 | PET-CT | DL | A&P | [66] |
| 27 | De Vos et al. | 2019 | Multiple | DL | A&P | [67] |
| 28 | Afridi et al. | 2016 | MRI | DL | Detection | [68] |
| 29 | Alansary et al. | 2019 | MRI | DL | Detection | [69] |
| 30 | Emad et al. | 2015 | MRI | DL | Detection | [13] |
| 31 | Goldfarb et al. | 2019 | MRI | DL | Detection | [70] |
| 32 | Kong et al. | 2016 | MRI | DL | Detection | [71] |
| 33 | Oksuz et al. | 2019 | MRI | DL | Detection | [5] |
| 34 | Baessler et al. | 2018 | MRI | ML-Non-DL | Detection | [72] |
| 35 | Baessler et al. | 2018 | MRI | ML-Non-DL | Detection | [73] |
| 36 | Kim et al. | 2017 | MRI | ML-Non-DL | Detection | [74] |
| 37 | Larroza et al. | 2018 | MRI | ML-Non-DL | Detection | [75] |
| 38 | Lorch et al. | 2017 | MRI | ML-Non-DL | Detection | [1] |
| 39 | Dormer et al. | 2018 | CT | DL | Detection | [15] |
| 40 | Fuhrman et al. | 2019 | CT | DL | Detection | [76] |
| 41 | Mannil et al. | 2018 | CT | Multiple | Detection | [77] |
| 42 | Wong et al. | 2016 | CT | Multiple | Detection | [78] |
| 43 | Ostvik et al. | 2019 | US | DL | Detection | [14] |
| 44 | Togo et al. | 2018 | PET-CT | Multiple | Detection | [79] |
| 45 | Bratanoff et al. | 2017 | Multiple | Other/Review | Detection | [80] |
| 46 | Howard et al. | 2019 | Other | DL | Detection | [11] |
| 47 | Singh et al. | 2018 | Other | DL | Detection | [10] |
| 48 | Margulis et al. | 2018 | Other | ML-Non-DL | Detection | [81] |
| 49 | Avendi et al. | 2016 | MRI | DL | Segmentation | [35] |
| 50 | Avendi et al. | 2016 | MRI | DL | Segmentation | [82] |
| 51 | Avendi et al. | 2017 | MRI | DL | Segmentation | [17] |
| 52 | Do et al. | 2019 | MRI | DL | Segmentation | [83] |
| 53 | Du et al. | 2019 | MRI | DL | Segmentation | [84] |
| 54 | Fahmy et al. | 2020 | MRI | DL | Segmentation | [85] |
| 55 | Hu et al. | 2019 | MRI | DL | Segmentation | [30] |
| 56 | Kim et al. | 2020 | MRI | DL | Segmentation | [86] |
| 57 | Leng et al. | 2018 | MRI | DL | Segmentation | [25] |
| 58 | Liu et al. | 2018 | MRI | DL | Segmentation | [28] |
| 59 | Ma et al. | 2019 | MRI | DL | Segmentation | [87] |
| 60 | Moccia et al. | 2019 | MRI | DL | Segmentation | [37] |
| 61 | Molaei et al. | 2017 | MRI | DL | Segmentation | [18] |
| 62 | Ngo et al. | 2017 | MRI | DL | Segmentation | [88] |
| 63 | Purmehdi et al. | 2019 | MRI | DL | Segmentation | [89] |
| 64 | Romaguera et al. | 2018 | MRI | DL | Segmentation | [27] |
| 65 | Tan et al. | 2017 | MRI | DL | Segmentation | [90] |
| 66 | Tan et al. | 2018 | MRI | DL | Segmentation | [91] |
| 67 | Tong et al. | 2019 | MRI | DL | Segmentation | [29] |
| 68 | Vigneault et al. | 2018 | MRI | DL | Segmentation | [92] |
| 69 | Wang et al. | 2019 | MRI | DL | Segmentation | [93] |
| 70 | Xiong et al. | 2019 | MRI | DL | Segmentation | [94] |
| 71 | Xu et al. | 2018 | MRI | DL | Segmentation | [95] |
| 72 | Yan et al. | 2019 | MRI | DL | Segmentation | [20] |
| 73 | Yang et al. | 2018 | MRI | DL | Segmentation | [96] |
| 74 | Yang et al. | 2019 | MRI | DL | Segmentation | [97] |
| 75 | Yang et al. | 2020 | MRI | DL | Segmentation | [98] |
| 76 | Yao et al. | 2018 | MRI | DL | Segmentation | [26] |
| 77 | Zheng et al. | 2018 | MRI | DL | Segmentation | [24] |
| 78 | Zotti et al. | 2019 | MRI | DL | Segmentation | [99] |
| 79 | Commandeur et al. | 2017 | CT | DL | Segmentation | [36] |
| 80 | Dormer et al. | 2018 | CT | DL | Segmentation | [100] |
| 81 | Gülsün et al. | 2016 | CT | DL | Segmentation | [101] |
| 82 | Jin et al. | 2018 | CT | DL | Segmentation | [102] |
| 83 | Lam et al. | 2019 | CT | DL | Segmentation | [103] |
| 84 | Lin et al. | 2019 | CT | DL | Segmentation | [16] |
| 85 | Wolterink et al. | 2019 | CT | DL | Segmentation | [12] |
| 86 | Sugiura et al. | 2013 | CT | ML-Non-DL | Segmentation | [104] |
| 87 | Arafati et al. | 2016 | US | DL | Segmentation | [32] |
| 88 | Leclerc et al. | 2019 | US | DL | Segmentation | [33] |
| 89 | Yu et al. | 2020 | US | DL | Segmentation | [105] |
| 90 | Wang et al. | 2019 | SPECT | DL | Segmentation | [34] |
| 91 | Morris et al. | 2019 | Multiple | DL | Segmentation | [106] |
| 92 | Bai et al. | 2018 | MRI | DL | Diagnosis | [39] |
| 93 | Bernard et al. | 2018 | MRI | DL | Diagnosis | [107] |
| 94 | Bratt et al. | 2018 | MRI | DL | Diagnosis | [108] |
| 95 | Du et al. | 2019 | MRI | DL | Diagnosis | [19] |
| 96 | Fahmy et al. | 2018 | MRI | DL | Diagnosis | [38] |
| 97 | Fahmy et al. | 2018 | MRI | DL | Diagnosis | [109] |
| 98 | Isensee et al. | 2018 | MRI | DL | Diagnosis | [21] |
| 99 | Khened et al. | 2019 | MRI | DL | Diagnosis | [22] |
| 100 | Lindsey et al. | 2020 | MRI | DL | Diagnosis | [110] |
| 101 | Luo et al. | 2018 | MRI | DL | Diagnosis | [111] |
| 102 | Ohta et al. | 2019 | MRI | DL | Diagnosis | [112] |
| 103 | Ruijsink et al. | 2018 | MRI | DL | Diagnosis | [41] |
| 104 | Ruijsink et al. | 2019 | MRI | DL | Diagnosis | [113] |
| 105 | Winther et al. | 2018 | MRI | DL | Diagnosis | [40] |
| 106 | Wu et al. | 2019 | MRI | DL | Diagnosis | [114] |
| 107 | Yuan et al. | 2019 | MRI | DL | Diagnosis | [115] |
| 108 | Zhang et al. | 2019 | MRI | DL | Diagnosis | [116] |
| 109 | Zhang et al. | 2019 | MRI | DL | Diagnosis | [117] |
| 110 | Zheng et al. | 2019 | MRI | DL | Diagnosis | [118] |
| 111 | Lei et al. | 2019 | MRI | ML-Non-DL | Diagnosis | [119] |
| 112 | Lungu et al. | 2016 | MRI | ML-Non-DL | Diagnosis | [120] |
| 113 | Mantilla et al. | 2015 | MRI | ML-Non-DL | Diagnosis | [121] |
| 114 | Satriano et al. | 2017 | MRI | ML-Non-DL | Diagnosis | [122] |
| 115 | Alis et al. | 2019 | MRI | Multiple | Diagnosis | [123] |
| 116 | Siegersma et al. | 2018 | MRI | Multiple | Diagnosis | [124] |
| 117 | Commandeur et al. | 2019 | CT | DL | Diagnosis | [125] |
| 118 | Commandeur et al. | 2019 | CT | DL | Diagnosis | [126] |
| 119 | Kumamaru et al. | 2019 | CT | DL | Diagnosis | [127] |
| 120 | Lessmann et al. | 2018 | CT | DL | Diagnosis | [44] |
| 121 | Martin et al. | 2019 | CT | DL | Diagnosis | [128] |
| 122 | Zreik et al. | 2018 | CT | DL | Diagnosis | [46] |
| 123 | Zreik et al. | 2019 | CT | DL | Diagnosis | [129] |
| 124 | Takx et al. | 2014 | CT | Multiple | Diagnosis | [45] |
| 125 | Beecy et al. | 2019 | US | DL | Diagnosis | [130] |
| 126 | Bienstock et al. | 2019 | US | DL | Diagnosis | [131] |
| 127 | Jafari et al. | 2019 | US | DL | Diagnosis | [43] |
| 128 | Komatsu et al. | 2019 | US | DL | Diagnosis | [132] |
| 129 | Kusunose et al. | 2018 | US | DL | Diagnosis | [133] |
| 130 | Kusunose et al. | 2019 | US | DL | Diagnosis | [134] |
| 131 | Silva et al. | 2018 | US | DL | Diagnosis | [135] |
| 132 | Ludomirsky et al. | 2018 | US | DL | Diagnosis | [136] |
| 133 | Madani et al. | 2018 | US | DL | Diagnosis | [137] |
| 134 | Matsuoka et al. | 2019 | US | DL | Diagnosis | [138] |
| 135 | Mazzanti et al. | 2015 | US | DL | Diagnosis | [139] |
| 136 | Moulson et al. | 2019 | US | DL | Diagnosis | [140] |
| 137 | Narang et al. | 2019 | US | DL | Diagnosis | [141] |
| 138 | Samtani et al. | 2019 | US | DL | Diagnosis | [142] |
| 139 | Sengupta et al. | 2016 | US | DL | Diagnosis | [143] |
| 140 | Shelby et al. | 2017 | US | DL | Diagnosis | [144] |
| 141 | Tablassian et al. | 2018 | US | ML-Non-DL | Diagnosis | [145] |
| 142 | Genovese et al. | 2019 | US | Other/Review | Diagnosis | [146] |
| 143 | Mahmoud et al. | 2017 | US | Other/Review | Diagnosis | [147] |
| 144 | Volpato et al. | 2019 | US | Other/Review | Diagnosis | [148] |
| 145 | Lee et al. | 2019 | US | Multiple | Diagnosis | [149] |
| 146 | Narula et al. | 2015 | US | Multiple | Diagnosis | [150] |
| 147 | Salem Omar et al. | 2018 | US | Multiple | Diagnosis | [151] |
| 148 | Case et al. | 2016 | PET-CT | DL | Diagnosis | [152] |
| 149 | Togo et al. | 2019 | PET-CT | DL | Diagnosis | [153] |
| 150 | DePuey et al. | 1989 | PET-CT | Other/Review | Diagnosis | [154] |
| 151 | Chiu et al. | 2019 | SPECT | DL | Diagnosis | [155] |
| 152 | Garcia et al. | 2018 | SPECT | DL | Diagnosis | [156] |
| 153 | Wang et al. | 2019 | SPECT | DL | Diagnosis | [34] |
| 154 | De Souza-Filho et al. | 2019 | SPECT | ML-Non-DL | Diagnosis | [157] |
| 155 | Puyol-Anton et al. | 2019 | Multiple | ML-Non-DL | Diagnosis | [158] |
| 156 | Domingos et al. | 2014 | Multiple | Other/Review | Diagnosis | [31] |
| 157 | Shade et al. | 2018 | Other | ML-Non-DL | Diagnosis | [159] |
| 158 | Curiale et al. | 2019 | MRI | DL | Diagnosis | [42] |
| 159 | Bello et al. | 2019 | MRI | DL | Prediction | [160] |
| 160 | Dawes et al. | 2017 | MRI | ML-Non-DL | Prediction | [161] |
| 161 | Samad et al. | 2018 | MRI | ML-Non-DL | Prediction | [162] |
| 162 | Coenen et al. | 2018 | CT | DL | Prediction | [163] |
| 163 | Commandeur et al. | 2019 | CT | DL | Prediction | [164] |
| 164 | Itu et al. | 2016 | CT | DL | Prediction | [165] |
| 165 | McElhinney et al. | 2019 | CT | DL | Prediction | [166] |
| 166 | Von Knebel et al. | 2019 | CT | DL | Prediction | [167] |
| 167 | Motwani et al. | 2016 | CT | ML-Non-DL | Prediction | [47] |
| 168 | Oikonomou et al. | 2019 | CT | Multiple | Prediction | [168] |
| 169 | Agasthya et al. | 2018 | US | ML-Non-DL | Prediction | [169] |
| 170 | Cikes et al. | 2019 | US | ML-Non-DL | Prediction | [170] |
| 171 | Eduardo et al. | 2019 | PET-CT | DL | Prediction | [171] |
| 172 | Juarez-Orozco et al. | 2019 | PET-CT | DL | Prediction | [172] |
| 173 | Juarez-Orozco et al. | 2018 | PET-CT | ML-Non-DL | Prediction | [173] |
| 174 | Betancur et al. | 2017 | SPECT | ML-Non-DL | Prediction | [174] |
| 175 | Betancur et al. | 2018 | SPECT | ML-Non-DL | Prediction | [175] |
| 176 | Hu et al. | 2018 | SPECT | ML-Non-DL | Prediction | [176] |
| 177 | Currie et al. | 2019 | Multiple | DL | Prediction | [177] |
| 178 | van Hamersvelt et al. | 2019 | Multiple | DL | Prediction | [178] |
| 179 | Ambale-Venkatesh | 2017 | Multiple | ML-Non-DL | Prediction | [179] |
| 180 | Rocon et al. | 2018 | Other | Multiple | Prediction | [180] |
| 181 | Aslan et al. | 2019 | MRI | DL | Other | [181] |
| 182 | Arafati et al. | 2019 | MRI | Other/Review | Other | [182] |
| 183 | Colletti et al. | 2019 | MRI | Other/Review | Other | [183] |
| 184 | Seetharam et al. | 2019 | MRI | Other/Review | Other | [184] |
| 185 | Seraphim et al. | 2019 | MRI | Other/Review | Other | [185] |
| 186 | Tao et al. | 2019 | MRI | Other/Review | Other | [186] |
| 187 | Gopalakrishnan et al. | 2015 | MRI | Multiple | Other | [187] |
| 188 | Nicol et al. | 2019 | CT | Other/Review | Other | [188] |
| 189 | Singh et al. | 2018 | CT | Other/Review | Other | [189] |
| 190 | Varga-Szemes et al. | 2018 | CT | Other/Review | Other | [190] |
| 191 | Luong et al. | 2016 | US | DL | Other | [191] |
| 192 | Narang et al. | 2019 | US | DL | Other | [192] |
| 193 | Cikes et al. | 2019 | US | ML-Non-DL | Other | [193] |
| 194 | Gandhi et al. | 2018 | US | Other/Review | Other | [194] |
| 195 | Garcia-Canadilla et al. | 2019 | US | Other/Review | Other | [195] |
| 196 | Al`Aref et al. | 2019 | Multiple | Other/Review | Other | [196] |
| 197 | Barone-Rochette et al. | 2019 | Multiple | Other/Review | Other | [197] |
| 198 | Commandeur et al. | 2018 | Multiple | Other/Review | Other | [198] |
| 199 | Cuocolo et al. | 2019 | Multiple | Other/Review | Other | [199] |
| 200 | Dey et al. | 2019 | Multiple | Other/Review | Other | [200] |
| 201 | Dilsizian et al. | 2014 | Multiple | Other/Review | Other | [201] |
| 202 | Dilsizian et al. | 2018 | Multiple | Other/Review | Other | [202] |
| 203 | Dorado-Díaz et al. | 2019 | Multiple | Other/Review | Other | [203] |
| 204 | Krittanawong et al. | 2019 | Multiple | Other/Review | Other | [204] |
| 205 | Leiner et al. | 2019 | Multiple | Other/Review | Other | [205] |
| 206 | Litjens et al. | 2017 | Multiple | Other/Review | Other | [206] |
| 207 | Litjens et al. | 2019 | Multiple | Other/Review | Other | [207] |
| 208 | Liu et al. | 2017 | Multiple | Other/Review | Other | [208] |
| 209 | Massalha et al. | 2018 | Multiple | Other/Review | Other | [209] |
| 210 | Mesanovic et al. | 2015 | Multiple | Other/Review | Other | [210] |
| 211 | O`Regan et al. | 2019 | Multiple | Other/Review | Other | [211] |
| 212 | Petersen et al. | 2019 | Multiple | Other/Review | Other | [212] |
| 213 | Retson et al. | 2019 | Multiple | Other/Review | Other | [213] |
| 214 | Seetharam et al. | 2019 | Multiple | Other/Review | Other | [214] |
| 215 | Shaw et al. | 2018 | Multiple | Other/Review | Other | [215] |
| 216 | Siegersma et al. | 2019 | Multiple | Other/Review | Other | [216] |
| 217 | Slomka et al. | 2017 | Multiple | Other/Review | Other | [217] |
| 218 | Strickland et al. | 2018 | Multiple | Other/Review | Other | [218] |
| 219 | Tsay et al. | 2018 | Multiple | Other/Review | Other | [219] |
| 220 | Wallis et al. | 2001 | Multiple | Other/Review | Other | [220] |
| 221 | Zhuang et al. | 2019 | Multiple | Other/Review | Other | [221] |
| 222 | Lyon et al. | 2019 | Multiple | Multiple | Other | [222] |
|  |  |  |  |  |  |  |
|  |  |  |  |  |  |  |
|  |  |  |  |  |  |  |

Abbreviations: DL = deep learning; ML-non-DL = machine learning techniques other than deep learning; A&P = image acquisition and preprocessing;

**Reference list**

1. Lorch B, Vaillant G, Baumgartner C, et al (2017) Automated Detection of Motion Artefacts in MR Imaging Using Decision Forests. J Med Eng 2017:1–9. https://doi.org/10.1155/2017/4501647

2. Lossau T, Nickisch H, Wissel T, et al (2019) Motion artifact recognition and quantification in coronary CT angiography using convolutional neural networks. Med Image Anal 52:68–79. https://doi.org/10.1016/j.media.2018.11.003

3. Küstner T, Armanious K, Yang J, et al (2019) Retrospective correction of motion-affected MR images using deep learning frameworks. Magn Reson Med 82:1527–1540. https://doi.org/10.1002/mrm.27783

4. Nakanishi R, Sankaran S, Grady L, et al (2018) Automated estimation of image quality for coronary computed tomographic angiography using machine learning. Eur Radiol 28:4018–4026. https://doi.org/10.1007/s00330-018-5348-8

5. Oksuz I, Ruijsink B, Puyol-Anton E, et al (2019) Automatic CNN-based detection of cardiac MR motion artefacts using k-space data augmentation and curriculum learning. Med Image Anal 55:136–147. https://doi.org/10.1016/j.media.2019.04.009

6. Tatsugami F, Higaki T, Nakamura Y, et al (2019) Deep learning–based image restoration algorithm for coronary CT angiography. Eur Radiol 29:5322–5329. https://doi.org/10.1007/s00330-019-06183-y

7. Qin C, Schlemper J, Caballero J, et al (2019) Convolutional recurrent neural networks for dynamic MR image reconstruction. IEEE Trans Med Imaging 38:280–290. https://doi.org/10.1109/TMI.2018.2863670

8. Schlemper J, Caballero J, Hajnal J V, et al (2018) A Deep Cascade of Convolutional Neural Networks for Dynamic MR Image Reconstruction. IEEE Trans Med Imaging 37:491–503. https://doi.org/10.1109/TMI.2017.2760978

9. Margeta J, Criminisi A, Cabrera Lozoya R, et al (2017) Fine-tuned convolutional neural nets for cardiac MRI acquisition plane recognition. Comput Methods Biomech Biomed Eng Imaging Vis 5:339–349. https://doi.org/10.1080/21681163.2015.1061448

10. Singh R, Kalra MK, Nitiwarangkul C, et al (2018) Deep learning in chest radiography: Detection of findings and presence of change. PLoS One 13:e0204155. https://doi.org/10.1371/journal.pone.0204155

11. Howard JP, Fisher L, Shun-Shin MJ, et al (2019) Cardiac Rhythm Device Identification Using Neural Networks. JACC Clin Electrophysiol 5:576–586. https://doi.org/10.1016/j.jacep.2019.02.003

12. Wolterink JM, van Hamersvelt RW, Viergever MA, et al (2019) Coronary artery centerline extraction in cardiac CT angiography using a CNN-based orientation classifier. Med Image Anal 51:46–60. https://doi.org/10.1016/j.media.2018.10.005

13. Emad O, Yassine IA, Fahmy AS, et al (2015) Automatic localization of the left ventricle in cardiac MRI images using deep learning. Conf Proc . Annu Int Conf IEEE Eng Med Biol Soc IEEE Eng Med Biol Soc Annu Conf 2015:683–686. https://doi.org/10.1109/EMBC.2015.7318454

14. Ostvik A, Smistad E, Aase SA, et al (2019) Real-Time Standard View Classification in Transthoracic Echocardiography Using Convolutional Neural Networks. Ultrasound Med Biol 45:374–384. https://doi.org/10.1016/j.ultrasmedbio.2018.07.024

15. Dormer JD, Halicek M, Ma L, et al (2018) Convolutional Neural Networks for the Detection of Diseased Hearts Using CT Images and Left Atrium Patches. Proc SPIE--the Int Soc Opt Eng 10575:. https://doi.org/10.1117/12.2293548

16. Lin H, Zou J, Li T, et al (2019) Development of a fast, multi-stage u-net for automatic segmentation of cardiac substructures in non-contrast ct images. Med Phys 46:e345. https://doi.org/10.1002/mp.13589

17. Avendi MR, Kheradvar A, Jafarkhani H (2017) Automatic segmentation of the right ventricle from cardiac MRI using a learning-based approach. Magn Reson Med 78:2439–2448. https://doi.org/10.1002/mrm.26631

18. Molaei S, Shiri M, Horan K, et al (2017) Deep Convolutional Neural Networks for left ventricle segmentation. Conf Proc . Annu Int Conf IEEE Eng Med Biol Soc IEEE Eng Med Biol Soc Annu Conf 2017:668–671. https://doi.org/10.1109/EMBC.2017.8036913

19. Du X, Yin S, Tang R, et al (2019) Cardiac-DeepIED: Automatic pixel-level deep segmentation for cardiac bi-ventricle using improved end-to-end encoder-decoder network. IEEE J Transl Eng Heal Med 7:1900110. https://doi.org/10.1109/JTEHM.2019.2900628

20. Yan W, Wang Y, van der Geest RJ, Tao Q (2019) Cine MRI analysis by deep learning of optical flow: Adding the temporal dimension. Comput Biol Med 111:103356. https://doi.org/10.1016/j.compbiomed.2019.103356

21. Isensee F, Jaeger PF, Full PM, et al (2018) Automatic Cardiac Disease Assessment on cine-MRI via Time-Series Segmentation and Domain Specific Features. Springer, Cham, pp 120–129

22. Khened M, Kollerathu VA, Krishnamurthi G, et al (2019) Fully convolutional multi-scale residual DenseNets for cardiac segmentation and automated cardiac diagnosis using ensemble of classifiers. Med Image Anal 51:21–45. https://doi.org/10.1016/j.media.2018.10.004

23. Luo B, Dong S, Wang K, et al (2018) Multi-views fusion CNN for left ventricular volumes estimation on cardiac MR images. IEEE Trans Biomed Eng 65:1924–1934. https://doi.org/10.1109/TBME.2017.2762762

24. Zheng Q, Delingette H, Duchateau N, et al (2018) 3-D Consistent and Robust Segmentation of Cardiac Images by Deep Learning With Spatial Propagation. IEEE Trans Med Imaging 37:2137–2148. https://doi.org/10.1109/TMI.2018.2820742

25. Leng S, Yang X, Zhao X, et al (2018) Computational Platform Based on Deep Learning for Segmenting Ventricular Endocardium in Long-axis Cardiac MR Imaging. Conf Proc . Annu Int Conf IEEE Eng Med Biol Soc IEEE Eng Med Biol Soc Annu Conf 2018:4500–4503. https://doi.org/10.1109/EMBC.2018.8513140

26. Yao H, Gryak J, Derksen H, et al (2018) Fully-automated left ventricle segmentation using a dilated and adversarial deep learning architecture. Circulation 138:

27. Romaguera LV, Romero FP, Fernandes Costa Filho CF, Fernandes Costa MG (2018) Myocardial segmentation in cardiac magnetic resonance images using fully convolutional neural networks. Biomed Signal Process Control 44:48–57. https://doi.org/10.1016/j.bspc.2018.04.008

28. Liu X, Shen Y, Zhang S, Zhao X (2018) Segmentation of left atrium through combination of deep convolutional and recurrent neural networks. J Med Imaging Heal Informatics 8:1578–1584. https://doi.org/10.1166/jmihi.2018.2511

29. Tong Q, Li C, Si W, et al (2019) RIANet: Recurrent interleaved attention network for cardiac MRI segmentation. Comput Biol Med 109:290–302. https://doi.org/10.1016/j.compbiomed.2019.04.042

30. Hu H, Pan N, Wang J, et al (2019) Automatic segmentation of left ventricle from cardiac MRI via deep learning and region constrained dynamic programming. Neurocomputing 347:139–148. https://doi.org/10.1016/j.neucom.2019.02.008

31. Domingos JS, Boardman H, Leeson P, Noble JA (2014) A new automated myocardial boundary delineator: Comparison of left ventricular volume by 3D echocardiography and cardiovascular magnetic resonance. Eur Heart J Cardiovasc Imaging 15:ii146. https://doi.org/10.1093/ehjci/jeu268

32. Arafati A, Avendi MR, Morisawa D, et al (2016) Fully automatic echocardiographic segmentation using machinelearning algorithms. J Am Soc Echocardiogr 29:B64

33. Leclerc S, Smistad E, Pedrosa J, et al (2019) Deep Learning for Segmentation Using an Open Large-Scale Dataset in 2D Echocardiography. IEEE Trans Med Imaging 38:2198–2210. https://doi.org/10.1109/TMI.2019.2900516

34. Wang T, Lei Y, Tang H, et al (2019) A learning-based automatic segmentation and quantification method on left ventricle in gated myocardial perfusion SPECT imaging: A feasibility study. J Nucl Cardiol. https://doi.org/10.1007/s12350-019-01594-2

35. Avendi MR, Kheradvar A, Jafarkhani H, et al (2016) A combined deep-learning and deformable-model approach to fully automatic segmentation of the left ventricle in cardiac MRI. Med Image Anal 30:108–119. https://doi.org/10.1016/j.media.2016.01.005

36. Commandeur F, Goeller M, Cadet S, et al (2017) Deep learning for fully automated cardiac segmentation and quantification of thoracic fat in non-contrast CT. J Cardiovasc Comput Tomogr 11:S39–S40. https://doi.org/https://doi.org/10.1016/j.compmedimag.2020.101717

37. Moccia S, Banali R, Martini C, et al (2019) Development and testing of a deep learning-based strategy for scar segmentation on CMR-LGE images. MAGMA 32:187–195. https://doi.org/10.1007/s10334-018-0718-4

38. Fahmy AS, Rausch J, Neisiusa U, et al (2018) Fully automated quantification of cardiac MR LV mass and scar in hypertrophic cardiomyopathy using deep learning. Circulation 138:

39. Bai W, Sinclair M, Tarroni G, et al (2018) Automated cardiovascular magnetic resonance image analysis with fully convolutional networks 08 Information and Computing Sciences 0801 Artificial Intelligence and Image Processing. J Cardiovasc Magn Reson 20:. https://doi.org/10.1186/s12968-018-0471

40. Winther HB, Hundt C, Schmidt B, et al (2018) ν-net: Deep Learning for Generalized Biventricular Mass and Function Parameters Using Multicenter Cardiac MRI Data. JACC Cardiovasc Imaging 11:1036–1038. https://doi.org/10.1016/j.jcmg.2017.11.013

41. Ruijsink JB, Puyol-Anton E, Sinclair M, et al (2018) Fully automated assessment of filling and ejection rates of the ventricle. Reference values for healthy volunteers from the UK-biobank cohort. Eur Heart J 39:876–877. https://doi.org/10.1093/eurheartj/ehy563.4382

42. Curiale AH, Colavecchia FD, Mato G, et al (2019) Automatic quantification of the LV function and mass: A deep learning approach for cardiovascular MRI. Comput Methods Programs Biomed 169:37–50. https://doi.org/10.1016/j.cmpb.2018.12.002

43. Jafari MH, Girgis H, N. VW, et al (2019) Automatic biplane left ventricular ejection fraction estimation with mobile point-of-care ultrasound using multi-task learning and adversarial training. Int J Comput Assist Radiol Surg 14:1027–1037. https://doi.org/10.1007/s11548-019-01954-w

44. Lessmann N, Van Ginneken B, Zreik M, et al (2018) Automatic Calcium Scoring in Low-Dose Chest CT Using Deep Neural Networks with Dilated Convolutions. IEEE Trans Med Imaging 37:615–625. https://doi.org/10.1109/TMI.2017.2769839

45. Takx RAP, de Jong PA, Leiner T, et al (2014) Automated Coronary Artery Calcification Scoring in Non-Gated Chest CT: Agreement and Reliability. PLoS One 9:e91239. https://doi.org/10.1371/journal.pone.0091239

46. Zreik M, Lessmann N, van Hamersvelt RW, et al (2018) Deep learning analysis of the myocardium in coronary CT angiography for identification of patients with functionally significant coronary artery stenosis. Med Image Anal 44:72–85. https://doi.org/10.1016/j.media.2017.11.008

47. Motwani M, Dey D, Berman DS, et al (2016) Machine learning for prediction of all-cause mortality in patients with suspected coronary artery disease: a 5-year multicentre prospective registry analysis. Eur Heart J 38:ehw188. https://doi.org/10.1093/eurheartj/ehw188

48. Bahrami N, Retson T, Blansit K, et al (2019) Automated selection of myocardial inversion time with a convolutional neural network: Spatial temporal ensemble myocardium inversion network (STEMI-NET). Magn Reson Med 81:3283–3291. https://doi.org/10.1002/mrm.27680

49. Biswas S, Aggarwal HK, Jacob M, et al (2019) Dynamic MRI using model-based deep learning and SToRM priors: MoDL-SToRM. Magn Reson Med 82:485–494. https://doi.org/10.1002/mrm.27706

50. Blansit K, Retson T, Masutani E, et al (2019) Deep learning-based prescription of cardiac MRI planes. Radiol Artif Intell 1:. https://doi.org/10.1148/ryai.2019180069

51. Chen Y, Shaw JL, Xie Y, et al (2019) Deep learning within a priori temporal feature spaces for large-scale dynamic MR image reconstruction: Application to 5-D cardiac MR Multitasking. Med Image Comput Comput Assist Interv 11765:495–504. https://doi.org/10.1007/978-3-030-32245-8_55

52. Fan L, Shen D, Haji-Valizadeh H, et al (2020) Rapid dealiasing of undersampled, non-Cartesian cardiac perfusion images using U-net. NMR Biomed e4239. https://doi.org/10.1002/nbm.4239

53. Ghodrati V, Shao J, Bydder M, et al (2019) MR image reconstruction using deep learning: Evaluation of network structure and loss functions. Quant Imaging Med Surg 9:1516–1527. https://doi.org/10.21037/qims.2019.08.10

54. Kofler A, Dewey M, Schaeffter T, et al (2019) Spatio-Temporal Deep Learning-Based Undersampling Artefact Reduction for 2D Radial Cine MRI with Limited Training Data. IEEE Trans Med Imaging. https://doi.org/10.1109/TMI.2019.2930318

55. Sandino C, Lai P, Vasanawala S, Cheng JY (2019) Deep learning-based reconstruction of 2D cardiac cine MRI data. Pediatr Radiol 49:S123. https://doi.org/10.1007/s00247-019-04376-7

56. Scannell CM, Veta M, Villa ADM, et al (2019) Deep-Learning-Based Preprocessing for Quantitative Myocardial Perfusion MRI. J Magn Reson Imaging. https://doi.org/10.1002/jmri.26983

57. Zhao C, Shao M, Carass A, et al (2019) Applications of a deep learning method for anti-aliasing and super-resolution in MRI. Magn Reson Imaging. https://doi.org/10.1016/j.mri.2019.05.038

58. Zhou Z, Han F, Ghodrati V, et al (2019) Parallel imaging and convolutional neural network combined fast MR image reconstruction: Applications in low-latency accelerated real-time imaging. Med Phys 46:3399–3413. https://doi.org/10.1002/mp.13628

59. Kim B, Han M, Shim H, et al (2019) A performance comparison of convolutional neural network-based image denoising methods: The effect of loss functions on low-dose CT images. Med Phys 46:3906–3923. https://doi.org/10.1002/mp.13713

60. Lossau (nee Elss) T, Nickisch H, Wissel T, et al (2019) Motion estimation and correction in cardiac CT angiography images using convolutional neural networks. Comput Med Imaging Graph 76:101640. https://doi.org/10.1016/j.compmedimag.2019.06.001

61. Lv T, Zhao W, Zhao Q, et al (2019) Deep learning-based dual-energy computed tomography imaging. Int J Comput Assist Radiol Surg 14:S98–S99. https://doi.org/10.1007/s11548-019-01969-3

62. Yan Z, Zhan Y, Peng Z, et al (2016) Multi-Instance Deep Learning: Discover Discriminative Local Anatomies for Bodypart Recognition. IEEE Trans Med Imaging 35:1332–1343. https://doi.org/10.1109/TMI.2016.2524985

63. Diller G-P, Lammers AE, Babu-Narayan S, et al (2019) Denoising and artefact removal for transthoracic echocardiographic imaging in congenital heart disease: utility of diagnosis specific deep learning algorithms. Int J Cardiovasc Imaging. https://doi.org/10.1007/s10554-019-01671-0

64. Fung A, Moulson N, Balthazaar S, et al (2019) Can Artificial Intelligence Assess Image Quality in Point-of-Care Ultrasound? Can J Cardiol 35:S146. https://doi.org/10.1016/j.cjca.2019.07.566

65. Karuzas A, Sablauskas K, Skrodenis L, et al (2019) Artificial intelligence in echocardiography - Steps to automatic cardiac measurements in routine practice. Eur Heart J 40:773. https://doi.org/10.1093/eurheartj/ehz748.0230

66. Ladefoged C, Hasbak P, Hansen J, et al (2019) Low-dose PET reconstruction using deep learning: Application to cardiac imaged with FDG. J Nucl Med 60:

67. de Vos BD, Berendsen FF, Viergever MA, et al (2019) A deep learning framework for unsupervised affine and deformable image registration. Med Image Anal 52:128–143. https://doi.org/10.1016/j.media.2018.11.010

68. Afridi MJ, Ross A, Liu X, et al (2016) Intelligent and automatic in vivo detection and quantification of transplanted cells in MRI. Mol Imaging Biol 18:S429–S430. https://doi.org/10.1007/s11307-016-1031-0

69. Alansary A, Oktay O, Li Y, et al (2019) Evaluating reinforcement learning agents for anatomical landmark detection. Med Image Anal 53:156–164. https://doi.org/10.1016/j.media.2019.02.007

70. Goldfarb JW, Craft J, Cao JJ, et al (2019) Water–fat separation and parameter mapping in cardiac MRI via deep learning with a convolutional neural network. J Magn Reson Imaging 50:655–665. https://doi.org/10.1002/jmri.26658

71. Kong B, Zhan Y, Shin M, et al (2016) Recognizing End-Diastole and End-Systole Frames via Deep Temporal Regression Network. Springer, Cham, pp 264–272

72. Baessler B, Mannil M, Oebel S, et al (2018) Subacute and chronic left ventricular myocardial scar: Accuracy of texture analysis on nonenhanced cine MR images. Radiology 286:103–112. https://doi.org/10.1148/radiol.2017170213

73. Baessler B, Mannil M, Maintz D, et al (2018) Texture analysis and machine learning of non-contrast T1-weighted MR images in patients with hypertrophic cardiomyopathy-Preliminary results. Eur J Radiol 102:61–67. https://doi.org/10.1016/j.ejrad.2018.03.013

74. Kim Y-C, Chung Y, Choe YH (2017) Automatic localization of anatomical landmarks in cardiac MR perfusion using random forests. Biomed Signal Process Control 38:370–378. https://doi.org/10.1016/j.bspc.2017.07.001

75. Larroza A, Lopez-Lereu MP, Monmeneu J V, et al (2018) Texture analysis of cardiac cine magnetic resonance imaging to detect nonviable segments in patients with chronic myocardial infarction. Med Phys 45:1471–1480. https://doi.org/10.1002/mp.12783

76. Fuhrman JD, Yip R, Henschke CI, et al (2019) Deep learning in the task of detecting coronary artery calcifications on low-dose thoracic CTs. Med Phys 46:e122. https://doi.org/10.1002/mp.13589

77. Mannil M, von Spiczak J, Manka R, Alkadhi H (2018) Texture Analysis and Machine Learning for Detecting Myocardial Infarction in Noncontrast Low-Dose Computed Tomography: Unveiling the Invisible. 53:338–343. https://doi.org/10.1097/RLI.0000000000000448

78. Wong KCL, Tee M, Chen M, et al (2016) Regional infarction identification from cardiac CT images: a computer-aided biomechanical approach. Int J Comput Assist Radiol Surg 11:1573–1583. https://doi.org/10.1007/s11548-016-1404-5

79. Togo R, Hirata K, Manabe O, et al (2018) Use of deep convolutional neural network-based features for detection of cardiac sarcoidosis from polar map. J Nucl Med 59:

80. Bratanoff M, Lin B, Ngernsritrakul T, et al (2017) Automated detection of mitral valve annular and leaflet geometry: Echocardiography and CT comparison. J Am Coll Cardiol 69:1580. https://doi.org/10.1016/S0735-1097(17)34969-0

81. Margulis K, Zhou Z, Fang Q, et al (2018) Combining Desorption Electrospray Ionization Mass Spectrometry Imaging and Machine Learning for Molecular Recognition of Myocardial Infarction. Anal Chem 90:12198–12206. https://doi.org/10.1021/acs.analchem.8b03410

82. Avendi MR, Kheradvar A, Jafarkhani H (2016) Fully automatic segmentation of heart chambers in cardiac MRI using deep learning. J Cardiovasc Magn Reson 18:. https://doi.org/https://doi.org/10.1186/1532-429X-18-S1-P351

83. Do HP, Guo Y, Yoon A., Nayak KS (2019) Accuracy, uncertainty, and adaptability of automatic myocardial ASL segmentation using deep CNN. Magn Reson Med. https://doi.org/10.1002/mrm.28043

84. Du X, Tang R, Yin S, et al (2019) Direct Segmentation-Based Full Quantification for Left Ventricle via Deep Multi-Task Regression Learning Network. IEEE J Biomed Heal Informatics 23:942–948. https://doi.org/10.1109/JBHI.2018.2879188

85. Fahmy AS, Neisius U, Chan RH, et al (2020) Three-dimensional deep convolutional neural networks for automated myocardial scar quantification in hypertrophic cardiomyopathy: A multicenter multivendor study. Radiology 294:52–60. https://doi.org/10.1148/radiol.2019190737

86. Kim Y-C, Kim KR, Choe YH (2020) Automatic myocardial segmentation in dynamic contrast enhanced perfusion MRI using Monte Carlo dropout in an encoder-decoder convolutional neural network. Comput Methods Programs Biomed 185:. https://doi.org/10.1016/j.cmpb.2019.105150

87. Ma Z, Wu X, Wang X, et al (2019) An iterative multi-path fully convolutional neural network for automatic cardiac segmentation in cine MR images. Med Phys 46:5652–5665. https://doi.org/10.1002/mp.13859

88. Ngo TA, Lu Z, Carneiro G (2017) Combining deep learning and level set for the automated segmentation of the left ventricle of the heart from cardiac cine magnetic resonance. Med Image Anal 35:159–171. https://doi.org/10.1016/j.media.2016.05.009

89. Purmehdi H, Hareendranathan AR, Noga M, Punithakumar K (2019) Right Ventricular Segmentation from MRI Using Deep Convolutional Neural Networks. Conf Proc . Annu Int Conf IEEE Eng Med Biol Soc IEEE Eng Med Biol Soc Annu Conf 2019:4020–4023. https://doi.org/10.1109/EMBC.2019.8857626

90. Tan LK, Liew YM, Lim E, et al (2017) Convolutional neural network regression for short-axis left ventricle segmentation in cardiac cine MR sequences. Med Image Anal 39:78–86. https://doi.org/10.1016/j.media.2017.04.002

91. Tan LK, McLaughlin RA, Lim E, et al (2018) Fully automated segmentation of the left ventricle in cine cardiac MRI using neural network regression. J Magn Reson Imaging 48:140–152. https://doi.org/10.1002/jmri.25932

92. Vigneault DM, Xie W, Ho CY, et al (2018) Ω-Net (Omega-Net): Fully automatic, multi-view cardiac MR detection, orientation, and segmentation with deep neural networks. Med Image Anal 48:95–106. https://doi.org/10.1016/j.media.2018.05.008

93. Wang Y, Xiong Z, Nalar A, et al (2019) A robust computational framework for estimating 3D Bi-Atrial chamber wall thickness. Comput Biol Med 114:103444. https://doi.org/10.1016/j.compbiomed.2019.103444

94. Xiong Z, Fedorov VV, Fu X, et al (2019) Fully Automatic Left Atrium Segmentation From Late Gadolinium Enhanced Magnetic Resonance Imaging Using a Dual Fully Convolutional Neural Network. IEEE Trans Med Imaging 38:515–524. https://doi.org/10.1109/TMI.2018.2866845

95. Xu C, Xu L, Gao Z, et al (2018) Direct delineation of myocardial infarction without contrast agents using a joint motion feature learning architecture. Med Image Anal 50:82–94. https://doi.org/10.1016/j.media.2018.09.001

96. Yang H, Sun J, Li H, et al (2018) Neural multi-atlas label fusion: Application to cardiac MR images. Med Image Anal 49:60–75. https://doi.org/10.1016/j.media.2018.07.009

97. Yang F, Zhang Y, Lei P, et al (2019) A Deep Learning Segmentation Approach in Free-Breathing Real-Time Cardiac Magnetic Resonance Imaging. Biomed Res Int 2019:5636423. https://doi.org/10.1155/2019/5636423

98. Yang F, Miao Y, Lei P, et al (2020) Development of a fully automatic segmentation method in cardiac magnetic resonance imaging using the deep learning approach. J Med Imaging Heal Informatics 10:11–17. https://doi.org/10.1166/jmihi.2020.2830

99. Zotti C, Luo Z, Lalande A, Jodoin P-M (2019) Convolutional Neural Network With Shape Prior Applied to Cardiac MRI Segmentation. IEEE J Biomed Heal informatics 23:1119–1128. https://doi.org/10.1109/JBHI.2018.2865450

100. Dormer JD, Ma L, Halicek M, et al (2018) Heart Chamber Segmentation from CT Using Convolutional Neural Networks. Proc SPIE--the Int Soc Opt Eng 10578:. https://doi.org/10.1117/12.2293554

101. Gülsün MA, Funka-Lea G, Sharma P, et al (2016) Coronary Centerline Extraction via Optimal Flow Paths and CNN Path Pruning. Springer, Cham, pp 317–325

102. Jin C, Feng J, Wang L, et al (2018) Left Atrial Appendage Segmentation Using Fully Convolutional Neural Networks and Modified Three-Dimensional Conditional Random Fields. IEEE J Biomed Heal informatics 22:1906–1916. https://doi.org/10.1109/JBHI.2018.2794552

103. Lam A, Cedilnik N, Vlachos K, et al (2019) Fully automated measurements of scar size on computed tomography relate to arrhythmia risk in post-infarction patients implanted with ICDs for primary prevention. Europace 21:ii974. https://doi.org/10.1093/europace/euz103

104. Sugiura T, Takeguchi T, Sakata Y, et al (2013) Automatic model-based contour detection of left ventricle myocardium from cardiac CT images. Int J Comput Assist Radiol Surg 8:145–155. https://doi.org/10.1007/s11548-012-0692-7

105. Yu X, Wang H, Ma L (2020) Ultrasound speckle tracking with deep convolutional neural network. J Med Imaging Heal Informatics 10:743–749. https://doi.org/10.1166/jmihi.2020.2927

106. Morris ED, Ghanem AI, Dong M, et al (2019) Cardiac substructure segmentation with deep learning for improved cardiac sparing. Med Phys 46:e257. https://doi.org/10.1002/mp.13940

107. Bernard O, Lalande A, Zotti C, et al (2018) Deep Learning Techniques for Automatic MRI Cardiac Multi-Structures Segmentation and Diagnosis: Is the Problem Solved? IEEE Trans Med Imaging 37:2514–2525. https://doi.org/10.1109/TMI.2018.2837502

108. Bratt A, Kim J, Beecy A, et al (2018) Deep learning for fully automated aortic valve flow quantification on phase velocity encoded cardiac magnetic resonance. Circulation 138:

109. Fahmy AS, Rausch J, Neisius U, et al (2018) Automated Cardiac MR Scar Quantification in Hypertrophic Cardiomyopathy Using Deep Convolutional Neural Networks. JACC Cardiovasc Imaging 11:1917–1918. https://doi.org/10.1016/j.jcmg.2018.04.030

110. Lindsey T, Lee J-J, T. L, et al (2020) Automated Cardiovascular Pathology Assessment Using Semantic Segmentation and Ensemble Learning. J Digit Imaging. https://doi.org/10.1007/s10278-019-00197-0

111. Luo Y (2017) Recurrent neural networks for classifying relations in clinical notes. J Biomed Inform 72:85–95. https://doi.org/10.1016/J.JBI.2017.07.006

112. Ohta Y, Yunaga H, Kitao S, et al (2019) Detection and classification of myocardial delayed enhancement patterns on mr images with deep neural networks: A feasibility study. Radiol Artif Intell 1:. https://doi.org/10.1148/ryai.2019180061

113. Ruijsink B, Puyol-Antón E, Oksuz I, et al (2019) Fully Automated, Quality-Controlled Cardiac Analysis From CMR: Validation and Large-Scale Application to Characterize Cardiac Function. JACC Cardiovasc Imaging. https://doi.org/10.1016/j.jcmg.2019.05.030

114. Wu J, Gan Z, Guo W, et al (2019) A fully convolutional network feature descriptor: Application to left ventricle motion estimation based on graph matching in short-axis MRI. Neurocomputing. https://doi.org/10.1016/j.neucom.2018.10.101

115. Yuan W-F, Zhao X-X, Hu F-B, et al (2019) Evaluation of Early Gadolinium Enhancement (EGE) and Cardiac Functional Parameters in Cine-Magnetic Resonance Imaging (MRI) on Artificial Intelligence in Patients with Acute Myocarditis: A Case-Controlled Observational Study. Med Sci Monit 25:5493–5500. https://doi.org/10.12659/MSM.916690

116. Zhang N, Yang G, Gao Z, et al (2019) Deep learning for diagnosis of chronic myocardial infarction on nonenhanced cardiac cine MRI. Radiology 291:606–607. https://doi.org/10.1148/radiol.2019182304

117. Zhang Q, Werys K, Lukaschuk E, et al (2019) Train the AI like a human observer: Deep learning with visualisation and guidance on attention in cardiac T1 mapping. Heart 105:A8–A9. https://doi.org/10.1136/heartjnl-2019-BSCMR.9

118. Zheng Q, Delingette H, Ayache N (2019) Explainable cardiac pathology classification on cine MRI with motion characterization by semi-supervised learning of apparent flow. Med Image Anal 56:80–95. https://doi.org/10.1016/j.media.2019.06.001

119. Lei L, Satriano A, Magyar-Ng M, et al (2019) Machine learning based automated diagnosis of ischemic vs non-ischemic dilated cardiomyopathy using 3D myocardial deformation analysis. Eur Heart J 40:3017. https://doi.org/10.1093/eurheartj/ehz746.0011

120. Lungu A, Swift AJ, Capener D, et al (2016) Diagnosis of pulmonary hypertension from magnetic resonance imaging-based computational models and decision tree analysis. Pulm Circ 6:181–190. https://doi.org/10.1086/686020

121. Mantilla J, Paredes J, Bellanger J-J, et al (2015) Classification of LV wall motion in cardiac MRI using kernel Dictionary Learning with a parametric approach. Conf Proc . Annu Int Conf IEEE Eng Med Biol Soc IEEE Eng Med Biol Soc Annu Conf 2015:7292–7295. https://doi.org/10.1109/EMBC.2015.7320075

122. Satriano A, Avitzur N, Wu C, et al (2017) Machine learning of three-dimensional left ventricular deformation for automated diagnostic support in amyloid, fabry, and hypertrophic cardiomyopathy: A cardiovascular MRI imaging study. Can J Cardiol 33:S23. https://doi.org/10.1016/j.cjca.2017.07.066

123. Alis D, Guler A, Yergin M, et al (2019) Assessment of ventricular tachyarrhythmia in patients with hypertrophic cardiomyopathy with machine learning-based texture analysis of late gadolinium enhancement cardiac MRI. Diagn Interv Imaging. https://doi.org/10.1016/j.diii.2019.10.005

124. Siegersma KR, Zreik M, Coroller T, et al (2018) Discrimination of fibrotic myocardium from healthy myocardium patients with aortic stenosis: A radiomics approach with machine learning models. Eur Heart J 39:971–972. https://doi.org/10.1093/eurheartj/ehy563.P4686

125. Commandeur F, Goeller M, Razipour A, et al (2019) Automated quantification of epicardial adipose tissue from non-contrast CT on multi-center and multi-vendor data using deep learning. Eur Heart J 40:3645. https://doi.org/10.1093/eurheartj/ehz746.0104

126. Commandeur F, Goeller M, Razipour A, et al (2019) Fully automated CT quantification of Epicardial adipose tissue by deep learning: A multicenter study. Radiol Artif Intell 1:. https://doi.org/10.1148/ryai.2019190045

127. Kumamaru KK, Fujimoto S, Otsuka Y, et al (2019) Diagnostic accuracy of 3D deep-learning-based fully automated estimation of patient-level minimum fractional flow reserve from coronary computed tomography angiography. Eur Heart J Cardiovasc Imaging. https://doi.org/10.1093/ehjci/jez160

128. Martin S, Fischer A, Van Assen M, et al (2019) Deep learning for automated calcium scoring in cardiac computed tomography. J Thorac Imaging 34:W62. https://doi.org/10.1097/RTI.0000000000000421

129. Zreik M, van Hamersvelt RW, Khalili N, et al (2019) Deep learning analysis of coronary arteries in cardiac CT angiography for detection of patients requiring invasive coronary angiography. IEEE Trans Med Imaging. https://doi.org/10.1109/TMI.2019.2953054

130. Beecy A, Bratt A, Brouwer L, et al (2019) Development of a Novel Deep Learning Model for Right Ventricular Quantification on Echocardiography. A Multimodality Validation Study. J Am Coll Cardiol 73:1437. https://doi.org/10.1016/S0735-1097(19)32043-1

131. Bienstock S, Samtani R, Liao S, et al (2019) Fully Automated Echocardiographic Artificial Intelligence Software Could Replace Contrast Agents for Improving Accuracy of Left Ventricular Ejection Fraction Quantification. J Am Soc Echocardiogr 32:B123–B124. https://doi.org/10.1016/j.echo.2019.04.414

132. Komatsu M, Matsuoka R, Sakaiu A, et al (2019) A novel deep learning based system for anomaly detection in fetal cardiac ultrasound screening. J Obstet Gynaecol Res 45:1702. https://doi.org/10.1111//jog.14030

133. Kusunose K, Abe T, Haga A, et al (2018) A deep learning approach for automated diagnosis of regional wall motion abnormality on echocardiography: A preliminary study. Circulation 138:. https://doi.org/https://doi.org/10.1016/j.jcmg.2019.02.024

134. Habib G, Erba PA, Iung B, et al (2019) Clinical presentation, aetiology and outcome of infective endocarditis. Results of the ESC-EORP EURO-ENDO (European infective endocarditis) registry: A prospective cohort study. Eur Heart J 40:3222-3232B. https://doi.org/10.1093/eurheartj/ehz620

135. Silva JM, Guerra A, Silva JF, et al (2018) CARS 2018—Computer Assisted Radiology and Surgery Proceedings of the 32nd International Congress and Exhibition Berlin, Germany, June 20–23, 2018. Int J Comput Assist Radiol Surg 13:1–273. https://doi.org/10.1007/s11548-018-1766-y

136. Ludomirsky A, Kezurer I, Beker S, Lipman Y (2018) Does artificial intelligence and deep learning architecture system accurately measure left ventricular systolic function? J Am Soc Echocardiogr 31:B103. https://doi.org/10.1016/j.echo.2018.04.010

137. Madani A, Ong JR, Tibrewal A, et al (2018) Deep echocardiography: data-efficient supervised and semi-supervised deep learning towards automated diagnosis of cardiac disease. npj Digit Med 1:59. https://doi.org/10.1038/s41746-018-0065-x

138. Matsuoka R, Komatsu M, Sakai S, et al (2019) A novel deep learning based system for segmental detection of normal fetal cardiac ultrasound movie. J Obstet Gynaecol Res 45:1660. https://doi.org/10.1111//jog.14030

139. Mazzanti M, Carlino F (2015) Artificial intelligence in cardiac imaging. Applications on hand held echo-preliminary data. Eur Heart J 36:253. https://doi.org/10.1093/eurheartj/ehv399

140. Moulson N, Fung A, Balthazaar S, et al (2019) Artificial Intelligence Assessment of Left Ventrcular Volumes and Function on POCUS Imaging. Can J Cardiol 35:S3–S4. https://doi.org/10.1016/j.cjca.2019.07.059

141. Narang A, Mor-Avi V, Prado A, et al (2019) Machine learning based automated dynamic quantification of left heart chamber volumes. Eur Heart J Cardiovasc Imaging 20:541–549. https://doi.org/10.1093/ehjci/jey137

142. Samtani R, Bienstock S, Liao S, et al (2019) Validation of a Novel Artificial Intelligence Left Ventricular Ejection Fraction Quantification Software (LVivoEF by DIA®) by Cardiac MRI. J Am Soc Echocardiogr 32:B119–B120. https://doi.org/10.1016/j.echo.2019.04.414

143. Sengupta PP, Huang Y-M, Bansal M, et al (2016) Cognitive Machine-Learning Algorithm for Cardiac Imaging: A Pilot Study for Differentiating Constrictive Pericarditis From Restrictive Cardiomyopathy. Circ Cardiovasc Imaging 9:. https://doi.org/10.1161/CIRCIMAGING.115.004330

144. Shelby A, Kelly CM, Li L, et al (2017) Comparison of traditional manual and fully automated methods of lv strain analysis in tetralogy of fallot. J Am Soc Echocardiogr 30:B23. https://doi.org/10.1016/j.echo.2017.04.007

145. Tabassian M, Sunderji I, Erdei T, et al (2018) Diagnosis of Heart Failure With Preserved Ejection Fraction: Machine Learning of Spatiotemporal Variations in Left Ventricular Deformation. J Am Soc Echocardiogr 31:1272-1284.e9. https://doi.org/10.1016/j.echo.2018.07.013

146. Genovese D, Rashedi N, Weinert L, et al (2019) Machine Learning–Based Three-Dimensional Echocardiographic Quantification of Right Ventricular Size and Function: Validation Against Cardiac Magnetic Resonance. J Am Soc Echocardiogr 32:969–977. https://doi.org/10.1016/j.echo.2019.04.001

147. Mahmoud A, Bansal M, Sengupta PP (2017) New Cardiac Imaging Algorithms to Diagnose Constrictive Pericarditis Versus Restrictive Cardiomyopathy. Curr Cardiol Rep 19:43. https://doi.org/10.1007/s11886-017-0851-0

148. Volpato V, Mor-Avi V, Narang A, et al (2019) Automated, machine learning-based, 3D echocardiographic quantification of left ventricular mass. Echocardiography 36:312–319. https://doi.org/10.1111/echo.14234

149. Lee S-Y, Ko T, Lee S-P (2019) Machine Learning Algorithm of Noninvasive Cardiac Imaging for Classification of Cardiac Mass. J Am Soc Echocardiogr 32:B119. https://doi.org/10.1016/j.echo.2019.04.414

150. Narula S, Khader S, Vallabhajosyula S, et al (2015) Automated morphological and functional phenotyping of human heart with feature tracking of 2D echocardiographic images using machine learning algorithms. J Am Soc Echocardiogr 28:B2

151. Salem Omar AM, Shameer K, Narula S, et al (2018) Artificial Intelligence-Based Assessment of Left Ventricular Filling Pressures From 2-Dimensional Cardiac Ultrasound Images. JACC Cardiovasc Imaging 11:509–510. https://doi.org/10.1016/j.jcmg.2017.05.003

152. Case PO, Bateman TM, Courter S, et al (2016) Using genetic artificial intelligence algorithms for determining optimal management of patients undergoing cardiac PET imaging. J Nucl Cardiol 23:917

153. Togo R, Hirata K, Manabe O, et al (2019) Cardiac sarcoidosis classification with deep convolutional neural network-based features using polar maps. Comput Biol Med 104:81–86. https://doi.org/10.1016/j.compbiomed.2018.11.008

154. DePuey EG, Garcia E V, Ezquerra NF (1989) Three-dimensional techniques and artificial intelligence in thallium-201 cardiac imaging. Am J Roentgenol 152:1161–1168. https://doi.org/10.2214/ajr.152.6.1161

155. Chiu C-H, Cheng C-Y, Tseng T-W, et al (2019) Deep learning analysis of Tl-201 myocardial perfusion imaging for improve diagnostic accuracy. J Nucl Med 60:

156. Garcia E V, Klein JL, Moncayo V, et al (2018) Diagnostic performance of an artificial intelligence-driven cardiac-structured reporting system for myocardial perfusion SPECT imaging. J Nucl Cardiol. https://doi.org/10.1007/s12350-018-1432-3

157. De Souza-Filho EM, De Amorim Fernandes F, Seixas FL, et al (2019) Artificial Intelligence tools for the evaluation of myocardial perfusion imaging. J Nucl Med 60:

158. Puyol-Anton E, Ruijsink B, Gerber B, et al (2019) Regional Multi-View Learning for Cardiac Motion Analysis: Application to Identification of Dilated Cardiomyopathy Patients. IEEE Trans Biomed Eng 66:956–966. https://doi.org/10.1109/TBME.2018.2865669

159. Okada DR, Smith J, Derakhshan A, et al (2018) Ventricular Arrhythmias in Cardiac Sarcoidosis. Circulation 138:1253–1264. https://doi.org/10.1161/CIRCULATIONAHA.118.034687

160. Bello GA, Dawes TJW, Duan J, et al (2019) Deep learning cardiac motion analysis for human survival prediction. Nat Mach Intell 1:95–104. https://doi.org/10.1038/s42256-019-0019-2

161. Dawes TJW, de Marvao A, Shi W, et al (2017) Machine Learning of Three-dimensional Right Ventricular Motion Enables Outcome Prediction in Pulmonary Hypertension: A Cardiac MR Imaging Study. Radiology 283:381–390. https://doi.org/10.1148/radiol.2016161315

162. Samad MD, Wehner GJ, Arbabshirani MR, et al (2018) Predicting deterioration of ventricular function in patients with repaired tetralogy of Fallot using machine learning. Eur Heart J Cardiovasc Imaging 19:730–738. https://doi.org/10.1093/ehjci/jey003

163. Coenen A, Kim Y-H, Kruk M, et al (2018) Diagnostic Accuracy of a Machine-Learning Approach to Coronary Computed Tomographic Angiography-Based Fractional Flow Reserve: Result From the MACHINE Consortium. Circ Cardiovasc Imaging 11:e007217. https://doi.org/10.1161/CIRCIMAGING.117.007217

164. Commandeur FC, Slomka PJ, Goeller M, et al (2019) Machine learning to predict the long-term risk of myocardial infarction and cardiac death based on clinical risk, coronary calcium and epicardial adipose tissue: A prospective study. Eur Heart J 40:4. https://doi.org/10.1093/eurheartj/ehz747.0002

165. Itu L, Rapaka S, Passerini T, et al (2016) A machine-learning approach for computation of fractional flow reserve from coronary computed tomography. J Appl Physiol 121:42–52. https://doi.org/10.1152/japplphysiol.00752.2015

166. McElhinney P, Eisenberg E, Commandeur F, et al (2019) Fully automated epicardial adipose tissue volume and density measured from non-contrast CT predict major adverse cardiovascular events in asymptomatic subjects. Eur Heart J 40:3763. https://doi.org/10.1093/eurheartj/ehz746.0757

167. von Knebel Doeberitz PL, De Cecco CN, Schoepf UJ, et al (2019) Impact of Coronary Computerized Tomography Angiography-Derived Plaque Quantification and Machine-Learning Computerized Tomography Fractional Flow Reserve on Adverse Cardiac Outcome. Am J Cardiol. https://doi.org/10.1016/j.amjcard.2019.07.061

168. Oikonomou EK, Williams MC, Kotanidis CP, et al (2019) A novel machine learning-derived radiotranscriptomic signature of perivascular fat improves cardiac risk prediction using coronary CTangiography. Eur Heart J 40:3529–3543. https://doi.org/10.1093/eurheartj/ehz592

169. Agasthya G, Jing L, Cerna AEU, et al (2018) Machine learning models show that global longitudinal strain is a strong predictor of survival after echocardiography that is superior to ejection fraction. Circulation 138:

170. Cikes M, Sanchez Martinez S, Claggett B, et al (2019) Machine-learning integration of complex echocardiographic patterns and clinical parameters from cohorts and trials. Eur Heart J 40:2549. https://doi.org/10.1093/eurheartj/ehz745.0147

171. Eduardo JOL, Knol RJJ, Octavio M-M, et al (2019) Deep learning in PET myocardial perfusion imaging: A study on cardiovascular event prediction. Rev Mex Cardiol 30:S9. https://doi.org/10.1016/j.jcmg.2019.08.009

172. Juarez-Orozco LE, Benjamins JW, Maaniitty T, et al (2019) Deep learning survival analysis enhances the value of hybrid PET/CT for long-term cardiovascular event prediction. Eur Heart J 40:675. https://doi.org/10.1093/eurheartj/ehz748.0177

173. Juarez-Orozco LE, Knol RJ, Martinez-Manzanera O, et al (2018) Identifying Mediate Adverse Events Through Deep Learning and PET Myocardial Perfusion Imaging. Glob Heart 13:373. https://doi.org/10.1016/j.gheart.2018.09.004

174. Betancur JA, Otaki Y, Fish M, et al (2017) Rest scan does not improve automatic machine learning prediction of major adverse coronary events after high speed myocardial perfusion imaging. J Am Coll Cardiol 69:1590. https://doi.org/10.1016/S0735-1097(17)34979-3

175. Betancur J, Otaki Y, Motwani M, et al (2018) Prognostic Value of Combined Clinical and Myocardial Perfusion Imaging Data Using Machine Learning. JACC Cardiovasc Imaging 11:1000–1009. https://doi.org/10.1016/j.jcmg.2017.07.024

176. Hu L-H, Betancur J, Sharir T, et al (2018) Machine learning predicts early coronary revascularization after fast myocardial SPECT: Results from multicenter REFINE SPECT registry. J Nucl Med 59:. https://doi.org/10.1093/ehjci/jez177

177. Currie G, Iqbal B, Kiat H (2019) Intelligent Imaging: Radiomics and Artificial Neural Networks in Heart Failure. J Med Imaging Radiat Sci 50:571–574. https://doi.org/10.1016/j.jmir.2019.08.006

178. van Hamersvelt RW, Isgum I, de Jong PA, et al (2019) Application of speCtraL computed tomogrAphy to impRove specIficity of cardiac compuTed tomographY (CLARITY study): rationale and design. BMJ Open 9:e025793. https://doi.org/10.1136/bmjopen-2018-025793

179. Ambale-Venkatesh B, Yang X, Wu CO, et al (2017) Cardiovascular Event Prediction by Machine Learning: The Multi-Ethnic Study of Atherosclerosis. Circ Res 121:1092–1101. https://doi.org/10.1161/CIRCRESAHA.117.311312

180. Rocon C, Tabassinan M, Tavares De Melo MD, et al (2018) Biventricular imaging markers to predict outcome in non-compaction cardiomyopathy: A machine learning study. Eur Heart J 39:1376–1377. https://doi.org/10.1093/eurheartj/ehy566.P6485

181. Aslan S, Hocke L, Schwarz N, Frederick B (2019) Extraction of the cardiac waveform from simultaneous multislice fMRI data using slice sorted averaging and a deep learning reconstruction filter. Neuroimage 198:303–316. https://doi.org/10.1016/j.neuroimage.2019.05.049

182. Arafati A, Hu P, Finn JP, et al (2019) Artificial intelligence in pediatric and adult congenital cardiac MRI: an unmet clinical need. Cardiovasc Diagn Ther 9:S310–S325. https://doi.org/10.21037/cdt.2019.06.09

183. Colletti PM (2019) Deep learning for cardiac MRI: The time has come. Radiology 290:89. https://doi.org/10.1148/radiol.2018182107

184. Seetharam K, Lerakis S (2019) Cardiac magnetic resonance imaging: the future is bright. F1000Research 8:. https://doi.org/10.12688/f1000research.19721.1

185. Seraphim A, Knott KD, Augusto J, et al (2019) Quantitative cardiac MRI. J Magn Reson Imaging. https://doi.org/10.1002/jmri.26789

186. Tao Q, Lelieveldt BPF, van der Geest RJ, et al (2019) Deep Learning for Quantitative Cardiac MRI. AJR Am J Roentgenol 1–7. https://doi.org/10.2214/AJR.19.21927

187. Gopalakrishnan V, Menon PG, Madan S (2015) cMRI-BED: A novel informatics framework for cardiac MRI biomarker extraction and discovery applied to pediatric cardiomyopathy classification. Biomed Eng Online 14:S7. https://doi.org/10.1186/1475-925X-14-S2-S7

188. Nicol ED, Norgaard BL, Blanke P, et al (2019) The Future of Cardiovascular Computed Tomography: Advanced Analytics and Clinical Insights. JACC Cardiovasc Imaging 12:1058–1072. https://doi.org/10.1016/j.jcmg.2018.11.037

189. Singh G, Al’Aref SJ, Van Assen M, et al (2018) Machine learning in cardiac CT: Basic concepts and contemporary data. J Cardiovasc Comput Tomogr 12:192–201. https://doi.org/10.1016/j.jcct.2018.04.010

190. Varga-Szemes A, Jacobs BE, Schoepf UJ (2018) The power and limitations of machine learning and artificial intelligence in cardiac CT. J Cardiovasc Comput Tomogr 12:202–203. https://doi.org/10.1016/j.jcct.2018.05.007

191. Luong C, Abdi A, Jue J, et al (2016) Automatic quality assessment of echo apical 4-chamber images using computer deep learning. Circulation 134:. https://doi.org/10.1109/TMI.2017.2690836.

192. Narang A, Hong H, Hsieh C, et al (2019) Evaluation of a Deep-Learning Algorithm Designed to Aid Novice Scanners in Obtaining Diagnostic Quality Echocardiograms. J Am Soc Echocardiogr 32:B118. https://doi.org/10.1016/j.echo.2019.04.414

193. Cikes M, Sanchez-Martinez S, Claggett B, et al (2019) Machine learning-based phenogrouping in heart failure to identify responders to cardiac resynchronization therapy. Eur J Heart Fail 21:74–85. https://doi.org/10.1002/ejhf.1333

194. Gandhi S, Mosleh W, Shen J, Chow C-M (2018) Automation, machine learning, and artificial intelligence in echocardiography: A brave new world. Echocardiography 35:1402–1418. https://doi.org/10.1111/echo.14086

195. Garcia-Canadilla P, Sanchez-Martinez S, Crispi F, et al (2019) Machine Learning in Fetal Cardiology: What to Expect. Fetal Diagn Ther 1–10. https://doi.org/10.1159/000505021

196. Al’Aref SJ, Anchouche K, Singh G, et al (2019) Clinical applications of machine learning in cardiovascular disease and its relevance to cardiac imaging. Eur Heart J 40:1975–1986. https://doi.org/10.1093/eurheartj/ehy404

197. Barone-Rochette G (2019) Will artificial intelligence change the job of the cardiac imaging specialist? Arch Cardiovasc Dis. https://doi.org/10.1016/j.acvd.2019.11.002

198. Commandeur M, Goeller M, Dey D (2018) Cardiac CT: Technological Advances in Hardware, Software, and Machine Learning Applications. Curr Cardiovasc Imaging Rep 11:. https://doi.org/10.1007/s12410-018-9459-z

199. Cuocolo R, Perillo T, De Rosa E, et al (2019) Current applications of big data and machine learning in cardiology. J Geriatr Cardiol 16:601–607. https://doi.org/10.11909/j.issn.1671-5411.2019.08.002

200. Dey D, Slomka PJ, Leeson P, et al (2019) Artificial Intelligence in Cardiovascular Imaging: JACC State-of-the-Art Review. J Am Coll Cardiol 73:1317–1335. https://doi.org/10.1016/j.jacc.2018.12.054

201. Dilsizian SE, Siegel EL (2014) Artificial intelligence in medicine and cardiac imaging: Harnessing big data and advanced computing to provide personalized medical diagnosis and treatment. Curr Cardiol Rep 16:441. https://doi.org/10.1007/s11886-013-0441-8

202. Dilsizian ME, Siegel EL (2018) Machine Meets Biology: a Primer on Artificial Intelligence in Cardiology and Cardiac Imaging. Curr Cardiol Rep 20:139. https://doi.org/10.1007/s11886-018-1074-8

203. Dorado-Diaz PI, Sampedro-Gomez J, Vicente-Palacios V, Sanchez PL (2019) Applications of Artificial Intelligence in Cardiology. The Future is Already Here. Rev Esp Cardiol 72:1065–1075. https://doi.org/10.1016/j.rec.2019.05.014

204. Krittanawong C, Johnson KW, Rosenson RS, et al (2019) Deep learning for cardiovascular medicine: a practical primer. Eur Heart J 40:2058–2073. https://doi.org/10.1093/eurheartj/ehz056

205. Leiner T, Rueckert D, Suinesiaputra A, et al (2019) Machine learning in cardiovascular magnetic resonance: basic concepts and applications. J Cardiovasc Magn Reson 21:61. https://doi.org/10.1186/s12968-019-0575-y

206. Litjens G, Kooi T, Bejnordi BE, et al (2017) A survey on deep learning in medical image analysis. Med Image Anal 42:60–88. https://doi.org/10.1016/j.media.2017.07.005

207. Litjens G, Ciompi F, Wolterink JM, et al (2019) State-of-the-Art Deep Learning in Cardiovascular Image Analysis. JACC Cardiovasc Imaging 12:1549–1565. https://doi.org/10.1016/j.jcmg.2019.06.009

208. Liu W, Wang Z, Liu X, et al (2017) A survey of deep neural network architectures and their applications. Neurocomputing 234:11–26. https://doi.org/10.1016/j.neucom.2016.12.038

209. Massalha S, Clarkin O, Thornhill R, et al (2018) Decision Support Tools, Systems, and Artificial Intelligence in Cardiac Imaging. Can J Cardiol 34:827–838. https://doi.org/10.1016/j.cjca.2018.04.032

210. Mesanovic N, Kusljugic Z (2015) Implementation of segmentation algorithms in cardiology. Anadolu Kardiyol Derg 15:90

211. O’Regan DP (2019) Putting machine learning into motion: applications in cardiovascular imaging. Clin Radiol 75:33–37. https://doi.org/10.1016/j.crad.2019.04.008

212. Petersen SE, Abdulkareem M, Leiner T (2019) Artificial Intelligence Will Transform Cardiac Imaging-Opportunities and Challenges. Front Cardiovasc Med 6:133. https://doi.org/10.3389/fcvm.2019.00133

213. Retson TA, Besser AH, Sall S, et al (2019) Machine learning and deep neural networks in thoracic and cardiovascular imaging. J Thorac Imaging 34:192–201. https://doi.org/10.1097/RTI.0000000000000385

214. Seetharam K, Shresthra S, Mills JD, Sengupta PP (2019) Artificial Intelligence in Nuclear Cardiology: Adding Value to Prognostication. Curr Cardiovasc Imaging Rep 12:. https://doi.org/10.1007/s12410-019-9490-8

215. Shaw LJ (2018) Can a Machine Learn Better Than Humans? JACC. Cardiovasc. Imaging 11:1010–1011

216. Siegersma KR, Leiner T, Chew DP, et al (2019) Artificial intelligence in cardiovascular imaging: state of the art and implications for the imaging cardiologist. Netherlands Hear J 27:403–413. https://doi.org/10.1007/s12471-019-01311-1

217. Slomka PJ, Betancur J, Liang JX, et al (2018) Rationale and design of the REgistry of Fast Myocardial Perfusion Imaging with NExt generation SPECT (REFINE SPECT). J Nucl Cardiol 1–12. https://doi.org/10.1007/s12350-018-1326-4

218. Strickland N (2018) What can Radiologists realistically expect from Artificial Intelligence? J Med Imaging Radiat Oncol 62:83. https://doi.org/10.1111/(ISSN)1754-9485

219. Tsay D, Patterson C (2018) From Machine Learning to Artificial Intelligence Applications in Cardiac Care. Circulation 138:2569–2575. https://doi.org/10.1161/CIRCULATIONAHA.118.031734

220. Wallis JW (2001) Invited commentary: Use of artificial intelligence in cardiac imaging. J Nucl Med 42:1192–1194

221. Zhuang X, Li L, Payer C, et al (2019) Evaluation of algorithms for Multi-Modality Whole Heart Segmentation: An open-access grand challenge. Med Image Anal 58:101537. https://doi.org/10.1016/j.media.2019.101537

222. Lyon A, Minchole A, Bueno-Orovio A, Rodriguez B (2019) Improving the clinical understanding of hypertrophic cardiomyopathy by combining patient data, machine learning and computer simulations: A case study. Morphologie. https://doi.org/10.1016/j.morpho.2019.09.001
